# Simultaneous knockout of multiple *LHCF* genes using single sgRNAs and engineering of a high‐fidelity Cas9 for precise genome editing in marine algae

**DOI:** 10.1111/pbi.13582

**Published:** 2021-04-09

**Authors:** Amit K. Sharma, Marianne Nymark, Snorre Flo, Torfinn Sparstad, Atle M. Bones, Per Winge

**Affiliations:** ^1^ Cell, Molecular Biology and Genomics Group Department of Biology Norwegian University of Science and Technology NTNU Trondheim Norway; ^2^ Present address: The University Centre in Svalbard UNIS Longyearbyen Norway

**Keywords:** Light‐harvesting complex proteins, High‐Fidelity (HiFi) Cas9 nuclease, precision genome editing, off‐target gene editing, *lhcf* mutants, diatom, *Phaeodactylum tricornutum*

## Abstract

The CRISPR/Cas9 system is an RNA‐guided sequence‐specific genome editing tool, which has been adopted for single or multiple gene editing in a wide range of organisms. When working with gene families with functional redundancy, knocking out multiple genes within the same family may be required to generate a phenotype. In this study, we tested the possibility of exploiting the known tolerance of Cas9 for mismatches between the single‐guide RNA (sgRNA) and target site to simultaneously introduce indels in multiple homologous genes in the marine diatom *Phaeodactylum tricornutum*. As a proof of concept, we designed two sgRNAs that could potentially target the same six light‐harvesting complex (LHC) genes belonging to the *LHCF* subgroup. Mutations in up to five genes were achieved simultaneously using a previously established CRISPR/Cas9 system for *P. tricornutum*. A visible colour change was observed in knockout mutants with multiple LHCF lesions. A combination of pigment, LHCF protein and growth analyses was used to further investigate the phenotypic differences between the multiple LHCF mutants and WT. Furthermore, we used the two same sgRNAs in combination with a variant of the existing Cas9 where four amino acids substitutions had been introduced that previously have been shown to increase Cas9 specificity. A significant reduction of off‐target editing events was observed, indicating that the altered Cas9 functioned as a high‐fidelity (HiFi) Cas9 nuclease.

## Introduction

Diatoms are unicellular photosynthetic microalgae found in aquatic environments that are responsible for approximately 40% of annual marine primary productivity, 20% of the global carbon fixation and oxygenation of the Earth’s atmosphere (Bowler *et al.,* 2010; Keiser *et al.,* 2011). In addition to the environmental and ecological importance of diatoms, they also have substantial biotechnological potential (Bozarth *et al.,* 2009; Kroth, 2007; Rotter *et al.,* 2021). Even though diatoms are considered as highly interesting candidates for the production of biofuels, fish feeds, biopharmaceuticals, antioxidants, foods, and several therapeutic supplements, yet they have only been commercially utilized to a lesser extent due to high production cost (Priyadarshani and Rath, 2012; Pulz and Gross, 2004; Rotter *et al.,* 2021; Wang and Seibert, 2017). Genetically optimized strains can potentially lower the production cost, and interest in genetic engineering of microalgae has increased in later years (Jeon *et al.,* 2017). Advancements in genetic engineering have produced molecular tools which can be used to modify genomes, overexpress and permanently modify the gene of interest in several algal species (Deng *et al.,* 2014; Radakovits *et al.,* 2010; Wei *et al.,* 2017; Weyman *et al.,* 2015). The recently discovered clustered regularly interspaced short palindromic repeats (CRISPR) technology has made targeted gene modification easier and cheaper and enabled the generation of knockout or knock‐in mutants (Hopes *et al.,* 2016; Nymark *et al.,* 2016; Shin *et al.,* 2016).

The CRISPR/CRISPR‐associated protein 9 (Cas9) system is a simple, cheap, accurate and highly efficient molecular technique for targeted genome modification. CRISPR/Cas9 has been successfully used to modify genes in a wide range of cells and organisms, including a few microalgae (Dicarlo *et al.,* 2013; Doudna and Charpentier, 2014; Hwang *et al.,* 2013; Nymark *et al.,* 2016; Yin *et al.,* 2014; Yu *et al.,* 2013). The Cas9 nuclease from *Streptococcus pyogenes* (SpCas9) forms a complex with a short single‐guide RNA (sgRNA) that determines the cutting site of the Cas9 nuclease. The Cas9‐sgRNA complex is guided to the target region simply by complementary base pairing between the first 20 nucleotides of the sgRNA and the targeted genomic DNA sequence, but the target region must be situated next to the protospacer adjacent motif (PAM) sequence NGG for cleavage of the DNA strand to take place (Doudna and Charpentier, 2014; Sander and Joung, 2014). The DNA region located 10–12 bp proximal to the PAM site is known as the seed region and is critical for base pairing and DNA cleavage. It is generally considered that mismatches in the seed region cannot be tolerated, while mismatches in the distal part (5′ of sgRNA) does not prevent Cas9 from cleaving DNA and therefore can generate off‐target mutations (Fu *et al.,* 2013; Hsu *et al.,* 2013; Kleinstiver *et al.,* 2016). These off‐target effects can complicate experimental research and limit the experimental use of CRISPR/Cas9. On the other hand, the off‐target effect can be an effective method to knock out multiple homologous and paralogous genes. Gene paralog redundancy is a common phenomenon observed in plants, animals and algae where several copies of a gene are generated through gene duplications events and often involves tandem gene duplications (De Martino *et al.,* 2000; Wang *et al.,* 2019). To obtain a knockout (KO) phenotype and understand the function of genes and proteins that are functionally redundant, it is often necessary to target several members of gene family. Generation of multiple KOs can be achieved by delivering a mixture of several Cas9 ribonucleoprotein particles (RNPs) (Seki and Rutz, 2018; Serif *et al.,* 2018), by co‐transformation of a Cas9 vector with an array of sgRNA expressing vectors (Mali *et al.,* 2013), co‐delivery of *in vitro* transcribed Cas9 mRNA and sgRNAs (Datsomor *et al.,* 2019; Yang *et al.,* 2013) and by delivering and expressing sgRNAs from a vector containing multiple sgRNA cassettes (Kabadi *et al.,* 2014). All these methods have limitations such as plasmid size, lower transformation efficiency, production cost, and being technically challenging to implement compared to a single plasmid‐based system. As a proof of concept to achieve multiple paralogous gene KOs by utilizing the off‐target effect of the CRISPR/Cas9 system in *P. tricornutum*, we chose to target a subgroup of genes within the large LHC family containing the main fucoxanthin (Fuco) chlorophyll (Chl) *a/c* proteins encoded by the *LHCF* genes (Büchel, 2015; Nymark *et al.,* 2013). In this study, we demonstrate that multiple genes can be knocked out using off‐target activities of Cas9 nuclease by successfully editing up to five *LHCF* genes.

SpCas9 is the most commonly used Cas9 variant for genome editing in plants, animals and microalgae. Off‐target editing is the result of pseudo‐specific interactions between Cas9 nuclease and unintended target regions (genomic loci that share a high degree of homology to the target site) which produce additional double‐strand breaks (DSBs) that might lead to undesired mutations. For most purposes, it is important that Cas9 selectively binds and induces mutations only at the intended region. Several methods have been developed and reported to reduce the off‐target effect associated with the SpCas9 nuclease in various organisms (Zhang, 2019). Off‐target effects of Cas9 have been reported in green algae (Baek *et al.,* 2016; Shin *et al.,* 2016), and we have observed re‐editing at the target site in cases where the first editing event resulted in only a small indel (1–2 bp) (Sharma *et al.,* 2018). Off‐target editing can be partly controlled with the design of the sgRNA. Enhanced specificity of SpCas9 has been achieved by modifying the guide RNA (Fu *et al.,* 2014). Several research groups have demonstrated increased specificity of Cas9 by limiting the amount of Cas9 either by delivering Cas9 mRNA or Cas9‐sgRNA RNP complexes (Cho *et al.,* 2014; Lin *et al.,* 2014). Similarly, the double‐nicking approach utilizing Cas9 nickase and two guide RNAs located side by side, tandem paired nicking, and rational engineering of Cas9 nuclease also significantly reduced off‐target events (Hyodo *et al.,* 2020; Nawaly *et al.,* 2020; Ran *et al.,* 2013). Furthermore, some methods require the expression of multiple sgRNAs and/or fusion of additional functional domains to Cas9, which can reduce the targeting range as well as create challenges for the delivery because of nucleic acid cargo size (Lino *et al.,* 2018; Pickar‐Oliver and Gersbach, 2019). Rational engineering of Cas9 has created high‐specificity variants, offering a simpler solution to minimize off‐target events without increasing the size of CRISPR/Cas9 components (Kleinstiver *et al.,* 2016; Slaymaker *et al.,* 2016). Furthermore, based on structural information and directed evolution, several research groups have made high‐fidelity (HiFi) variants of Cas9 that exhibits off‐target effects at undetectable levels (Zhang, 2019).

Based on the structure of SpCas9 and sgRNA, Kleinstiver *et al.,* (2016) generated a modified SpCas9 that retained the specificity and efficiency for the on‐target site while reducing the off‐target activity to undetectable levels. A HiFi Cas9 variant (SpCas9‐HF1) (Kleinstiver *et al.,* 2016) and enhanced specificity SpCas9 (eSpCas9) (Slaymaker *et al.,* 2016) variants were created by substituting four amino acids. These Cas9 variants were predicted to have lower binding energy for target site recognition and cleavage, but sufficient binding energy to achieve cleavage. Based on their findings, we substituted the same four amino acids in the Cas9 used for editing of *P. tricornutum* (diaCas9) as in SpCas9‐HF1 to test whether we could achieve higher specificity. The modified HiFi‐diaCas9 has the same catalytic domain as SpCas9‐HF1. We then compared the modified HiFi‐diaCas9 with the standard diaCas9 using the same sgRNAs that target the highly homologous *LHCF* genes and found that in contrast with the standard diaCas9, HiFi‐diaCas9 had substantially lower off‐target activity.

## Result and discussion

### *LHCF* target genes

**Figure 1:**
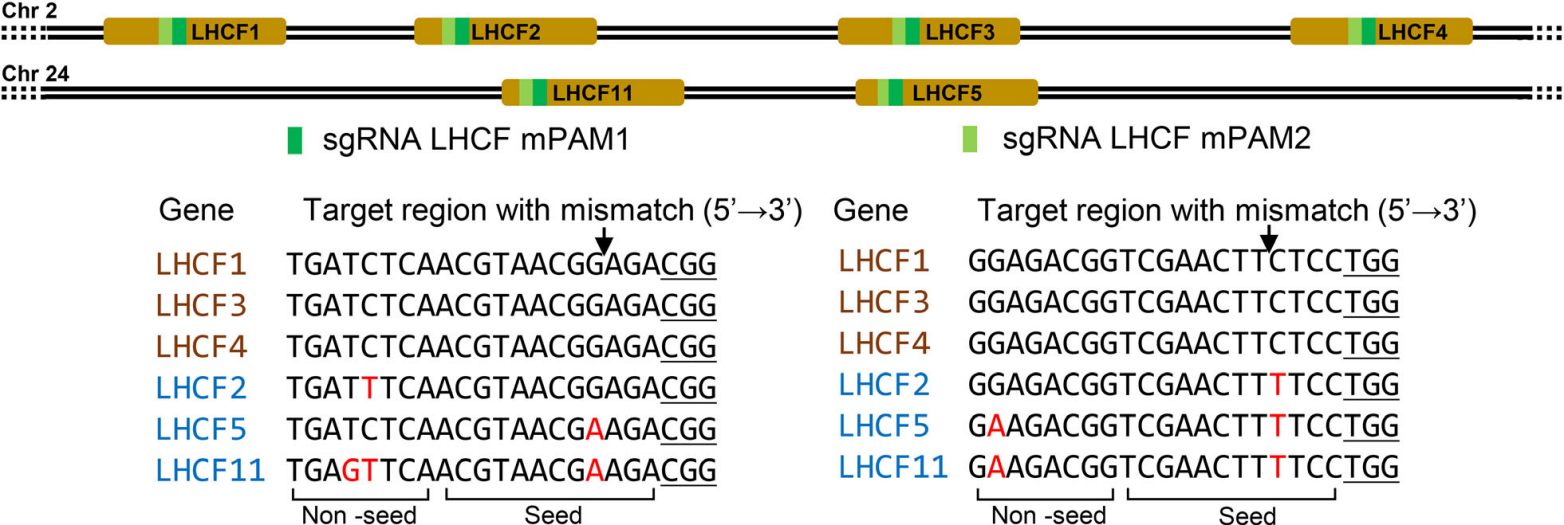
Overview of *LHCF* genes and target sequences of *LHCF* mPAM1 and mPAM2 sgRNAs. The localization of targeted *LHCF* genes in chromosome 2 and 24 is schematically presented. Three predicted on target (*LHCF1, LHCF3, LHCF4*) regions and one target region with mismatch (*LHCF2*) are located in chromosome 2, whereas two possible target regions with mismatches (*LHCF5* and *LHCF11*) are located in chromosome 24. The target sites of LHCF mPAM1 and LHCF mPAM2 are highlighted with dark green and light green rectangles, respectively. Brown font colour of genes names before the target region indicates target sites without mismatches, whereas blue font colour indicates target sites with mismatches. Mismatched nucleotides are labelled red. PAM sites (NGG) are underlined. Cas9 cut site at the target region is indicated by a black arrow. More information about target regions is given in Table S1.

The *LHCF* gene family has 17 members in *P. tricornutum* (Bowler *et al.,* 2008; Büchel, 2015; Nymark *et al.,* 2013) and shows a high degree of sequence similarity both at the DNA and protein level (Figure S1) (Kilian *et al.,* 2011). In order to test the possibility and efficiency of mutating multiple homologous genes simultaneously, we designed two sgRNAs called LHCF mPAM1 and LHCF mPAM2 that both had the potential to target the same six *LHCF* genes. Both sgRNAs have 20 bp perfect match with target sites in the *LHCF1*, *LHCF3* and *LHCF4* genes and display 1–3 mismatches between the sgRNA sequences and the target sites in the *LHCF2*, *LHCF5* and *LHCF11* genes as shown in Figure 1 and Table S1.

### Generation of multiple *LHCF* mutants using CRISPR/Cas9 in *P. tricornutum*

The pKS diaCas9_sgRNA plasmid (Nymark *et al.,* 2016) expressing either LHCF mPAM1 or LHCF mPAM2 sgRNA was delivered to *P. tricornutum* cells by biolistic particle bombardment. Approx. 50%–70% of the primary colonies were found to contain a fragment of the diaCas9 gene (Figure S2). Cas9‐positive primary colonies were analysed for on‐target mutations in the *LHCF1* gene. Sequence analysis of the *LHCF1* PCR products showed that around half of the primary colonies resulting from transformation with the pKS diaCas9_sgRNA plasmids contained mutations at the *LHCF1* target site. The high percentage of on‐target mutations achieved with both sgRNAs used is consistent with previous studies (Nymark *et al.,* 2016; Sharma *et al.,* 2018). Cells from cultures grown from primary colonies containing mutations in the *LHCF1* gene were re‐plated to obtain colonies originating from single cells (secondary colonies) since each primary colony may be a heterogeneous mixture of cells with mutated and non‐mutated *lhcf* alleles.

### On‐target analyses of CRISPR/Cas9‐induced mutations

Eleven secondary colonies (six from LHCF mPAM1 and five from LHCF mPAM2 sgRNAs) derived from primary colonies confirmed to harbour mutations in *LHCF1* were selected for targeted mutation analysis. DiaCas9‐mediated cleavage had taken place at all three on‐target loci (*LHCF1, LHCF3* and *LHCF4*) with high efficiency (on average 90%; 10 out of 11 lines) for both sgRNAs (Table 1). In 50% of the mutant cells, Sanger sequencing revealed that only one allele had been amplified by PCR, indicating loss of heterozygosity (loss of allelic polymorphism) (Kosicki *et al.,* 2018). An explanation for the loss of allelic polymorphism could be homologous recombination or the effect of chromothripsis producing long scrambled pieces of DNA at the target sites or large indels (George *et al.,* 2020; Zhang *et al.,* 2015). We considered a gene to be edited when a mutagenic event could be confirmed in both alleles (biallelic) and when only one mutated allele could be detected as long as no WT allele was present (monoallelic detection).

**Table 1:** Overview of multiple gene editing events achieved using the CRISPR/Cas9 system with diaCas9 in combination with LHCF mPAM1 or LHCF mPAM2 sgRNAs. Genes edited are denoted as ‘√’ and non‐edited as “X”. Presence of either monoallelic (loss of polymorphism where only one allele was detected with no trace of WT allele) or biallelic mutation is considered as edited. On‐target regions (containing no mismatches with sgRNA) are labelled green while off‐target regions (containing mismatches with sgRNA) are labelled orange. The number of mismatched nucleotides is indicated below the gene names. Target regions not amplifiable by PCR are also considered as edited. Results presented here are from secondary colonies and the tertiary colony 6.1.11 [Correction added on 24 August 2021, after first online publication: Colour shading has been updated in this version for Tables 1 and 3].

	*LHCF1* Mismatch; mPAM1: 0 mPAM2: 0		*LHCF3* Mismatch; mPAM1: 0 mPAM2: 0	*LHCF4* Mismatch; mPAM1: 0 mPAM2: 0	*LHCF2* Mismatch; mPAM1: 1 mPAM2: 1	*LHCF5* Mismatch; mPAM1: 1 mPAM2: 2	*LHCF11* Mismatch; mPAM1: 3 mPAM2: 2
**LHCF mPAM1**							
Colony 3.1	**√**	**√**		**X**	**√**	**X**	**X**
Colony 6.1.11	**√**	**√**		**√**	**√**	**√**	**X**
Colony 6.4	**√**	**√**		**√**	**√**	**√**	**X**
Colony 15.1	**√**	**√**		**√**	**√**	**√**	**X**
Colony 5.1	**√**	**√**		**√**	**√**	**√**	**X**
Colony 1.10	**√**	**√**		**√**	**√**	**X**	**X**
Edited/non‐edited	**6/6**	**6/6**		**5/6**	**6/6**	**4/6**	**0/6**
**LHCF mPAM2**							
Colony 3.1	**√**	**√**		**√**	**√**	**X**	**X**
Colony 5.4	**√**	**√**		**√**	**√**	**X**	**X**
Colony 8.1	**√**	**√**		**√**	**√**	**X**	**X**
Colony 8.5	**√**	**√**		**√**	**√**	**X**	**X**
Colony 10.1	**√**	**X**		**√**	**√**	**X**	**X**
Edited/non‐edited	**5/5**	**5/5**		**5/5**	**5/5**	**0/5**	**0/5**

### Off‐target analyses of CRISPR/Cas9‐induced mutations

Previous studies have shown that SpCas9 can induce off‐target editing at unwanted genomic locations when the guide RNA has three or fewer mismatches with the target sequence (Fu *et al.,* 2013; Hsu *et al.,* 2013). In diatoms, it has been reported that the diaCas9 system can re‐edit the target region if the first gene editing event produces 1–2 bp indels and there is a positive selection for the corrected WT allele (Sharma *et al.,* 2018). However, whole‐genome re‐sequencing of a mutant generated using the same Cas9 nuclease could not detect mutations at the predicted off‐target sites (Russo *et al.,* 2018). To assess the targeting specificity of diaCas9, all mutants containing indels at intended target sites were examined for off‐target editing. Potential off‐target sites were predicted by our in‐house Perl‐based programme and the online CRISPR design tools for both sgRNAs, in total six for LHCF mPAM1 and five for LHCF mPAM2 (Table S1). Among the computationally predicted off‐target sites, *LHCF* genes with 1–3 bp mismatches were assessed for off‐target DNA cleavage by PCR and sequencing.

The PCR and sequencing data showed that all secondary clones contained indels at off‐target locus *LHCF2* (mismatch = 1, non‐seed region) and four out of six mutants contained off‐target indels at *LHCF5* (mismatch = 1, seed region) when using the LHCF mPAM1 sgRNA. Similarly, five out of five secondary clones contained off‐target mutations in *LHCF2* (mismatch = 1, seed region) when paired with LHCF mPAM2 sgRNA (Table 1). However, no off‐target indels were detected at the *LHCF5* target site where there were 2 bp mismatches with the LHCF mPAM2 sgRNA and one of the mismatches was in the seed region (Figure 1, Table S1). No indels were detected at the target site in *LHCF11* (mismatch = 2–3) for either of the two sgRNAs (Table 1). Several previous studies show that mismatches in the seed region (the 10–12 base pairs adjacent to the PAM) are critical and determine the specificity of Cas9 compared to the distal part of the sgRNA sequence (non‐seed sequence) (Hsu *et al.,* 2013; Kim *et al.,* 2013; Pattanayak *et al.,* 2013). In contrast with previous reports, mutations were detected in all LHCF mPAM2 mutants in the *LHCF2* gene possessing 1 bp mismatch in the seed region (Figure 1; four bp 5′ upstream of the PAM site). Similarly, four out of six LHCF mPAM1 mutants contained mutations in the *LHCF5* gene possessing 1 bp mismatch in the seed region. From these data, it can be concluded that in our system 1 bp mismatch in the seed region of the guide RNA (i.e. 10–12 bases before the PAM) may not be enough to avoid off‐target effects induced by CRISPR/Cas9 if the rest of the guide RNA has 100% match. The sequence beyond the seed region also confers target specificity, but 1–2 mismatches might be tolerated (Jao *et al.,* 2013). In the present study, 2 bp mismatches were enough to abolish the off‐target effect when one of the mismatches was located in the seed region. This finding is consistent with studies in human cells and other model organisms (Manghwar *et al.,* 2020; Fu *et al.,* 2013; Hsu *et al.,* 2013; Naeem *et al.,* 2020). Additionally, several studies have reported that specificity of Cas9 is determined by several other factors such as sgRNA structures (Hsu *et al.,* 2013), an effective concentration of the Cas9_sgRNA complex (Wu *et al.,* 2014), and GC content of the sgRNA (Doench *et al.,* 2014). Off‐target mutations are a major concern as it can complicate experimental research and limit the experimental use of CRISPR/Cas9 systems. These off‐target effects can be avoided by careful design of the sgRNA. However, off‐target effects of the Cas9 nuclease may be useful for disrupting multiple homologous or duplicated genes using the sgRNA CRISPR‐Cas9 system.

### Multiple *LHCF* gene KOs result in pigmentation and growth phenotypes

**Figure 2:**
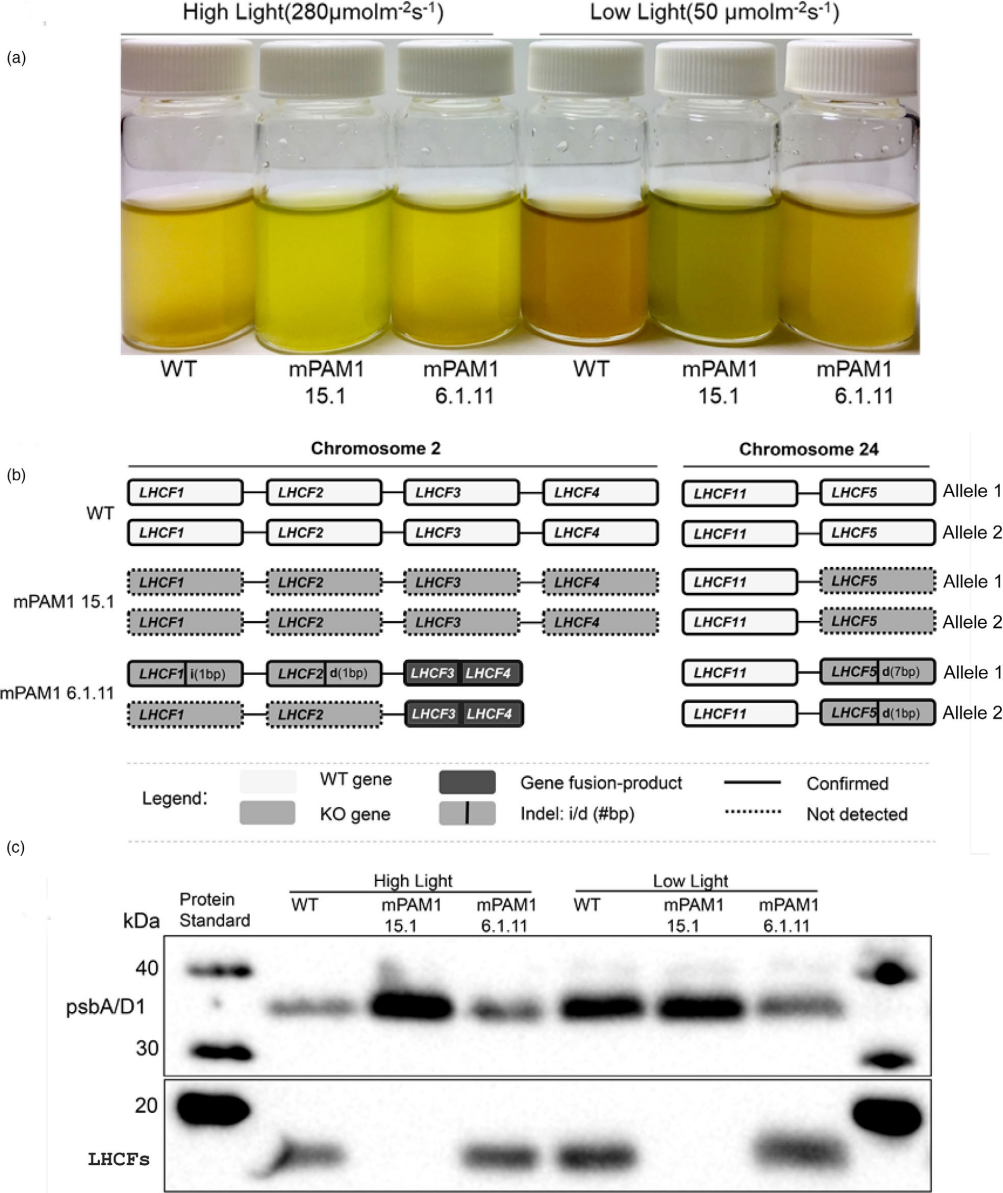
Disruption of multiple *LHCF* genes. (a) Phenotypes of high light (280 µmol photons/m^2^/s, HL) and low light (50 µmol photons/m^2^/s, LL) acclimated *P. tricornutum* WT, *lhcf* mPAM1 15.1 and *lhcf* mPAM1 6.1.11 cultures. All cell cultures were adjusted to equal cell density (27 million cells/mL) and imaged with an iPhone 6s. (b) Schematic overview of the genetic composition of mutated *LHCF* genes in *lhcf* mPAM1 15.1 and *lhcf* mPAM1 6.1.11 mutants. WT genes are white. Knocked out (KO) genes are light grey and annotated by indel (i/d) and the base length of the indel (#bp) if known. In‐frame gene fusion products are labelled dark grey. Confirmed DNA sequences are labelled with a solid border. Target regions that could not be amplified by PCR have dashed borders. (c) Western blot analysis of LHCFs proteins from WT and *lhcf* mPAM1 mutant lines acclimated to HL or LL conditions. *P. tricornutum* WT, *lhcf* mPAM1 mutants 15.1 and 6.1.11 were grown for two weeks in HL and LL before harvesting and isolation of total protein. Proteins separated on an SDS‐PAGE gel were blotted on a nitrocellulose membrane, and polyclonal rabbit antibodies were used to target LHCFs, and D1‐protein (psbA/D1) of photosystem II. MagicMarkTM XP Western Protein standards report the protein mass (in kDa).

We selected two of the multiple *lhcf* KO mutants based on preliminary sequencing data and a changed coloration compared to WT cells for further investigations. These mutants were generated using diaCas9 coupled with LHCF mPAM1 sgRNA. Editing was possible at on‐target sites *LHCF1*, *LHCF3* and *LHCF4*, and off‐target sites containing 1 bp mismatch (*LHCF2*, *LHCF5*). Based on PCR and Sanger sequencing, these lines contained indels in multiple LHCF genes. The *LHCF1*, *LHCF2* and *LHCF5* genes were knocked out in *lhcf* mPAM1 6.1.11. In addition, this line contained an in‐frame gene fusion of *LHCF3* and *LHCF4* (see Figure S3 for DNA and protein sequence). The second line (*lhcf* mPAM1 15.1) was a quintuple *lhcf* KO where we were unable to amplify and detect *LHCF1‐5* by PCR (Figure 2B, Figure S3). A gene expression analysis of the *lhcf* mPAM1 15.1 line showed that no transcripts were detected for *LHCF1‐5* genes, while *LHCF11* was slightly up‐regulated (Figure S4). We also noticed a slight up‐regulation of LHCR1 and LHCR2. Genes located upstream of *LHCF1*, *Phatr2_43334* and downstream of *LHCF4*, *Phatr2_43326* were expressed close to WT levels, with expression of *Phatr2_43326* slightly reduced (Figure S4). Gene expression results from the *lhcf* mPAM1 6.1.11 line were similar with low or no expression of *LHCF1*, *LHCF2* and *LHCF5* and a slightly reduced expression of the *LHCF3‐4* fusion gene (Figure S4). The strongly reduced expression of *LHCF1*, *LHCF2* and *LHCF5*, which all contain alleles with frameshift indels, is most likely due to premature stop codons in the transcripts. This may sometimes activate the non‐sense‐mediated decay pathway (NMD) and lead to reduced transcript levels (Hug *et al.,* 2016).

**Figure 3:**
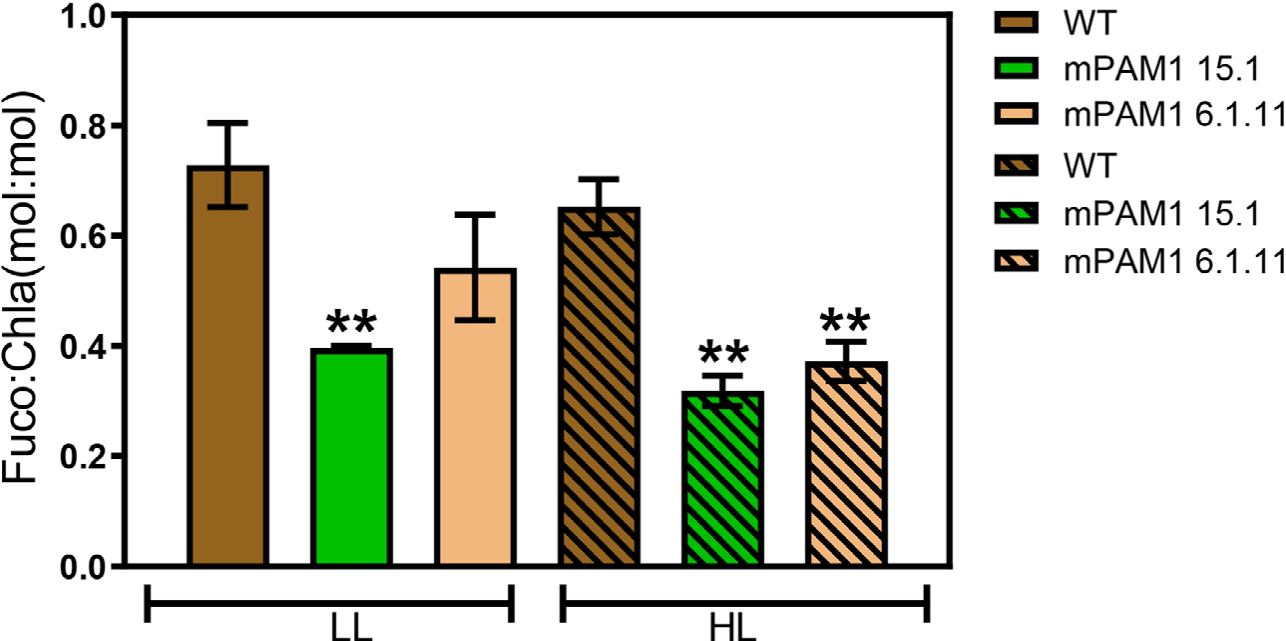
Effects of knocking out multiple *LHCF* genes at pigment level. WT and multiple *lhcf* mutants acclimated to HL (280 µmol photons/m^2^/s) and LL (50 µmol photons/m^2^/s). Fuco per Chl *a* (Fuco:Chl *a*) ratios are presented as the mean of n replicates of the relative unit mol:mol, with the standard error of the mean (±SEM, nWT = 9, n6.1.11 = 4, n15.1 = 4). Statistical significance was inferred using two‐sided t‐test assuming heteroskedastic variance. Asterisk (**) denotes significant difference with *P* < 0.001.

To investigate the effects of knocking out multiple *LHCF* genes, the two mutant lines, the WT and a phenotypically normally looking control line *lhcf* mPAM1 1.10, were grown under low light (LL; 50 µmol photons/m^2^/s) and high light (HL; 280 µmol photons/m^2^/s). Differences in the coloration of the cell cultures, level of LHCF proteins, pigment content and growth were examined in cultures acclimated to the two different light conditions. The multiple *lhcf* KO mutants displayed changes in coloration compared to WT (Figure 2a) and the *lhcf* mPAM1 1.10 control line (Figure S5A). In *lhcf* mPAM1 1.10, both alleles of *LHCF1* are knocked out. In addition, one of the *LHCF2* alleles is inactivated and there is a perfect in‐frame fusion of the *LHCF3* and *LHCF4* genes resulting in no changes in the protein sequence. Mutations in *LHCF1*, *LHCF2* and *LHCF5* in *lhcf* mPAM1 6.1.11 resulted in a lighter brown colour, whereas the additional loss of *LHCF3 and LHCF4* in *lhcf* mPAM1 15.1 resulted in a green coloration (Figure 2a). A similar green coloration as for the *lhcf* mPAM1 15.1 mutant has previously been reported in mutants of *P. tricornutum ALB3b* showing a 75% decrease in LHCF protein level (Nymark *et al.,* 2019). In line *lhcf* mPAM1 1.10, where only the *LHCF1* gene is biallelically knocked out, no difference in coloration was observed. To confirm the loss of LHCF proteins in the multiple *lhcf* KO lines, Western blot analyses were performed with an antibody predicted to bind to a highly conserved epitope of the LHCF1‐11 proteins (Juhas et al., 2014). No band was visible for the *lhcf* mPAM1 15.1 line, whereas the band detected for *lhcf* mPAM1 6.1.11 was similar to WT (Figure 2c). These results indicate that the LHCF1‐11 antibody might primarily detect the highly abundant LHCF3‐4 proteins and confirm that LHCF3‐4 are not present in *lhcf* mPAM1 15.1. LHCF protein expression might be slightly reduced in *lhcf* mPAM1 1.10 under both LL and HL conditions compared to WT cells (Figure S5), possibly due to loss of *LHCF1*, inactivation of one allele of *LHCF2* and *LHCF3‐4* gene fusion. Fuco is responsible for the golden brown colour characteristic of diatoms, and their ability to absorb light of the green waveband. This carotenoid is only present in the LHC complexes, whereas Chl *a* is present in both the LHC complexes and the photosystems. As expected by the observed colour changes in the mutant cultures, the pigment analyses confirmed that the Fuco:Chl *a* ratio was lower in *lhcf* multiple KO mutants than in WT and *lhcf* mPAM1 1.10, indicating that multiple *lhcf* KOs result in a smaller light‐harvesting antenna size (Figure 3; Figure S5D). The Fuco:Chl *a* ratio decreased with increased LHCF perturbance. The greenish colour of the *lhcf* mPAM1 15.1 culture might also indicate additional structural changes in the antenna since the absorption properties of Fuco strongly depends on the protein environment (Gundermann and Büchel, 2014; Wang *et al.,* 2019). The *lhcf* mPAM1 15.1 mutant showed retarded cell growth in both LL and HL compared to WT cells (Table 2, Figure S6A‐B).

**Table 2:** Proliferation rate of WT and two *lhcf* mutants in high light (HL) and low light (LL) conditions. Maximum division per day and doubling time (h) are presented with mean value and standard error of the mean (SE, *n* = 3). Proliferation rate was calculated from the exponential phase of the growth curve

	Division per day	±SE	Doubling time(h)	±SE
WT LL	2.14	0.03	11.20	0.18
mPAM1 15.1 LL	1.60	0.10	14.55	0.95
mPAM1 6.1.11 LL	2.36	0.04	10.37	0.16
WT HL	2.16	0.02	11.16	0.11
mPAM1 15.1 HL	1.40	0.03	17.19	0.32
mPAM1 6.1.11 HL	2.02	0.09	12.06	0.57

In contrast, the cell growth of *lhcf* mPAM1 6.1.11 mutant was not significantly affected and grew similar to WT cells in both light conditions (Table 2, Figure S6A‐B). Fit and healthy cells retain a highly functional photosynthetic apparatus and has high maximum quantum yield of PSII photochemistry (Φ_PSIImax_) (Bourge and Frankignoulle, 2000). Φ_PSIImax_ was low in *lhcf* mPAM1 15.1 in both LL and HL, whereas *lhcf* mPAM1 6.1.11 showed Φ_PSIImax_ values similar to WT levels (Figure S6C‐D). A distortion of the antenna structure in the *lhcf* mPAM1 15.1 mutant causing less efficient transferal of energy to the photosystems could explain the large differences in growth rate between the mutants. Combined these data show that diaCas9 LHCF mPAM1 successfully induced mutations at several LHCF loci resulting in phenotypic differences compared to WT.

### Efficiency and specificity of HiFi‐diaCas9‐sgRNA

**Figure 4:**
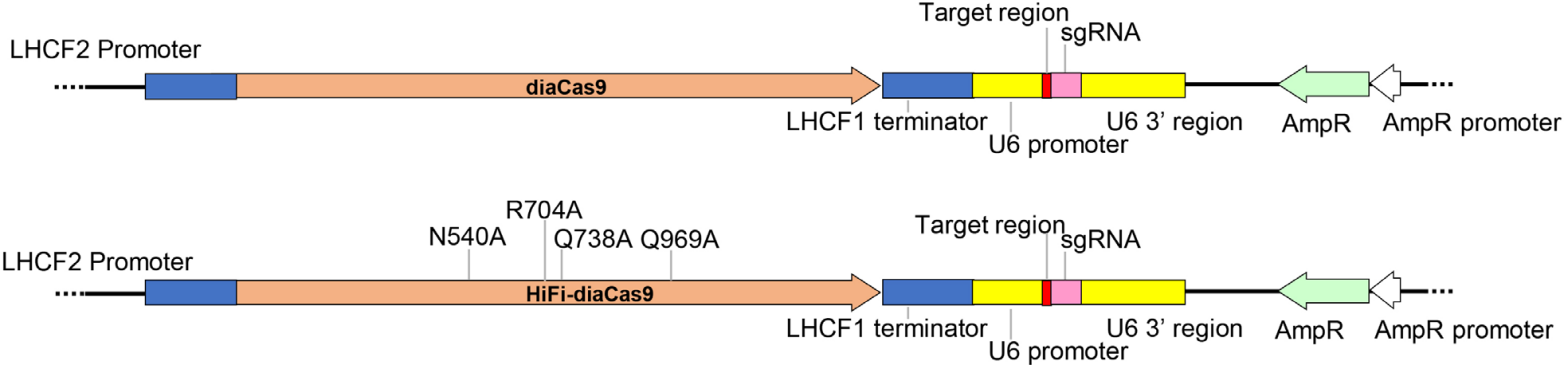
Schematic representation of CRISPR/Cas9 vectors used in this study. Standard diaCas9 (top, pKS diaCas9_sgRNA) and HiFi‐diaCas9 (bottom, HiFi‐pKS diaCas9_sgRNA) vectors differ in four amino acid residue positions. The substitutions are indicated in the HiFi‐diaCas9 gene. The U6 promoter drives sgRNA expression while expression of Cas9 protein is driven by the *LHCF2* promoter.

Several different strategies have been used to increase the specificity of SpCas9 like reducing the exposure time to the active nucleases, optimizing the sgRNA, developing inducible CRISPR/Cas systems and amino acid modifications of Cas nucleases (Pickar‐Oliver and Gersbach, 2019; Zhang, 2019). Although none of these methods are fool proof, rational engineering of Cas9 has proven to be a more promising and simple method to solve specificity problems (Kleinstiver *et al.,* 2016; Slaymaker *et al.,* 2016). In order to increase the specificity of standard diaCas9 (expressed from pKS diaCas9_sgRNA), we substituted the same four amino acids (N540A, R704A, Q738A and Q969A) (Figure 4) as in the study by Kleinstiver *et al*. (2016). These amino acids form direct hydrogen bonds with the phosphate backbone of the target DNA strand, and their modification is thought to increase the specificity of Cas9 by reducing the excess binding energy associated with the Cas9‐sgRNA/DNA complex (Kleinstiver *et al.,* 2016). This mutated version of diaCas9 was named High‐fidelity diatom Cas9 (HiFi‐diaCas9). To determine the editing efficiency and specificity of HiFi‐diaCas9, we performed direct comparisons with standard diaCas9 using the same sgRNAs (LHCF mPAM1 and LHCF mPAM2) that introduced various kinds of indels at on‐ and off‐target genomic locus when paired with standard diaCas9.

### Moderate decrease in on‐target editing efficiency of Hifi‐diaCas9 in secondary colonies

On‐target DNA editing efficiency at the *LHCF1* target site of primary colonies confirmed to contain a fragment of Cas9, was found to be similar for both Cas9 variants (Table 3 and Figure S2).

**Table 3:** Overview of genes edited utilizing combinations of LHCF mPAM1 or LHCF mPAM2 sgRNAs with standard diaCas9 and HiFi‐diaCas9. Detected editing are labelled ‘√’ and non‐editing ‘X’. On‐target mutations are labelled green, while off‐target mutations are labelled orange. Presence of either monoallelic (loss of polymorphism where only one allele was detected with no trace of WT allele) or biallelic mutation is considered as edited. *LHCF* target sites are accompanied with the number of mismatched nucleotides between sgRNA and DNA target sequence. Editing efficiency (number of edited colonies/total number of colonies analysed) is indicated below each target site. Numbers of mutated *LHCF* genes in individual colonies are indicated in the rightmost column (on‐target (black) and off‐target (red)). Overall editing efficiency of diaCas9 and HiFi‐diaCas9 is calculated by combining all target sites for both LHCF mPAM sgRNAs

**Cas9**	**sgRNA**	**Mutant strain**	**LHCF1** Mismatch; mPAM1:0 mPAM2: 0	**LHCF3** Mismatch; mPAM1:0 mPAM2: 0	**LHCF4** Mismatch; mPAM1:0 mPAM2: 0	**LHCF2** Mismatch; mPAM1:1 mPAM2: 1	**LHCF5** Mismatch; mPAM1:1 mPAM2: 2	**Number of Mutated LHCF** On‐target, off‐target
diaCas9	mPAM1	3.1	**√**	**√**	**×**	**√**	**×**	**2, 1**
		6.1.11	**√**	**√**	**√**	**√**	**√**	**3, 2**
		6.4	**√**	**√**	**√**	**√**	**√**	**3, 2**
		15.1	**√**	**√**	**√**	**√**	**√**	**3, 2**
		1.10	**√**	**√**	**√**	**√**	**×**	**3, 1**
		5.1	**√**	**√**	**√**	**√**	**√**	**3, 2**
	mPAM2	3.1	**√**	**√**	**√**	**√**	**×**	**3, 1**
		5.4	**√**	**√**	**√**	**√**	**×**	**3, 1**
		8.1	**√**	**√**	**√**	**√**	**×**	**3, 1**
		8.5	**√**	**√**	**√**	**√**	**×**	**3, 1**
		10.1	**√**	**√**	**√**	**√**	**×**	**3, 1**
		**Editing efficiency**	**11/11 [100%]**	**11/11 [100%]**	**10/11 [90%]**	**11/11 [100%]**	**4/11 [36%]**	
		**Overall efficiency**	**32/33 [96.6%]**			**15/22 [68%]**		
HiFi‐diaCas9	mPAM1	3.1	**×**	**√**	**√**	**×**	**×**	**2, 0**
		5.1	**√**	**√**	**√**	**×**	**×**	**3, 0**
		14.2	**√**	**×**	**×**	**×**	**×**	**1, 0**
		15.2	**×**	**√**	**√**	**×**	**×**	**2, 0**
	mPAM2	2.1	**√**	**√**	**×**	**√**	**×**	**2, 1**
		4.1	**√**	**√**	**×**	**×**	**×**	**2, 0**
		6.1	**√**	**√**	**×**	**×**	**×**	**2, 0**
		6.5	**√**	**√**	**√**	**×**	**×**	**3, 0**
		8.5	**√**	**√**	**×**	**×**	**×**	**2, 0**
		**Editing efficiency**	**7/9 [77%]**	**8/9 [88%]**	**4/9 [44%]**	**1/9 [11%]**	**0/9 [0%]**	
		**Overall efficiency**	**19/27 [71%]**			**1/18 [5%]**		

To compare the efficiency of HiFi‐diaCas9 with standard diaCas9 more comprehensively, we isolated secondary colonies from primary colonies with confirmed *LHCF1* mutations. A subset of secondary colonies was selected and screened for indels at all three on‐target genomic locations by PCR followed by Sanger sequencing. We found that HiFi‐diaCas9 exhibited on‐target activities that were 25% lower than what was observed with standard diaCas9 for LHCF mPAM1 and similar for two out of three on‐target sites for LHCF mPAM2 (Table 3, Figure S7). The editing efficiency was low when LHCF mPAM2 sgRNA was used in combination with HiFi‐diaCas9 to target *LHCF4*. Only one of five clones was edited when using HiFi‐diaCas9 compared to six out of six for diaCas9 (Table 3). Combined assessment of on‐target nuclease activities tested with two sgRNAs revealed that alanine substitutions at four residues moderately reduce the on‐target cleavage efficiency of HiFi‐diaCas9 compared to standard diaCas9 (Table 3). Experiments with additional sgRNAs need to be performed to be able to reach a conclusion regarding the editing efficiency of the HiFi‐diaCas9 compared to standard diaCas9.

### HiFi‐diaCas9‐sgRNA shows lower off‐target editing

We next sought to determine whether HiFi‐diaCas9 could reduce mutagenic events at predicted off‐target sites that were observed to be edited by standard diaCas9. Examination of predicted off‐target regions showed that none of the HiFi‐diaCas9_LHCF mPAM1 sgRNA derived mutant lines contained indels at the predicted off‐target genomic locus *LHCF2* as opposed to standard diaCas9_LHCF mPAM1 sgRNA derived mutant lines (Table 3, Figure S7). Similarly, no off‐target events were detected in *LHCF5* when HiFi‐diaCas9_LHCF mPAM1 sgRNA had been introduced into the cells, whereas a mutation was detected in four out of six diaCas9_LHCF mPAM1 sgRNA lines for this gene (Table 3, Figure S7). Only one out of five HiFi‐diaCas9_LHCF mPAM2 sgRNA derived mutants showed editing at the *LHCF2* target site compared to five out of five diaCas9_LHCF mPAM2 sgRNA derived mutants (Table 3, Figure S7). No mutations were detected in *LHCF11* where numbers of mismatches were three for LHCF mPAM1 and two for LHCF mPAM2 when using either of the two Cas9 versions. Similarly, no mutations were detected in *LHCF5* independently of which Cas9 version that were used when targeted with LHCF mPAM2 sgRNA. Here, the number of mismatches between the sgRNA and *LHCF5* was two and one of the mismatches was located in the seed region (Figure 1, Table 3, Figure S7). Overall, *LHCF1‐5* off‐target activity for diaCas9 was found to be around 45% while for HiFi‐diaCas9 it was around 3% (Table 3). The combined data indicate that HiFi‐diaCas9 has a higher specificity than the standard diaCas9. The lower off‐target activity is expected due to a predicted reduced binding energy between Cas9 and target DNA strands. Similar findings were reported by Kleinstiver and co‐workers (Kleinstiver *et al.,* 2016) where substituting four amino acids reduced the on‐target activities but could not completely eliminate off‐target effects. The somewhat lowered on‐target activities can be accepted if high specificity is required.

### Conclusion

We have shown that the off‐target activity of diaCas9 can be exploited to create multiple KOs of genes with high sequence similarity and that multiple *LHCF* gene KOs were necessary to obtain clear phenotypic differences compared to WT. We have also created a HiFi‐diaCas9 enzyme that retained approximately 70% of the on‐target activity reported for diaCas9 and that only caused an editing event in one out of 18 tested off‐target sites. In contrast, diaCas9 was highly promiscuous, and introduced DSBs in all cell lines at the 1 bp mismatch off‐target site of *LHCF2*. Hence, it is argued that the HiFi‐Cas9 enzyme may provide the fidelity that diaCas9 lacks, especially when targeting DNA sequences with high similarity to other genomic loci.

## Material and methods

### Construction of the HiFi‐diaCas9_sgRNA vector

The HiFi‐diaCas9 was constructed by replacing a 2505 bp region of the Cas9 with a custom designed synthetic DNA fragment (GeneArt, Thermo Fisher Scientific) that contained the following four amino substitutions: N540A, R704A, Q738A and Q969A. BsrGI and BSiWI restriction enzyme cutting sites were included at the 5′ and 3′ of the DNA fragment, respectively, to facilitate cloning. The HiFi‐diaCas9 fragment was inserted into the diaCas9_sgRNA vector (Nymark *et al.,* 2016) by digesting both the HiFi‐diaCas9 fragment and the diaCas9_sgRNA vector with BsrGI‐HF and BSiWI restriction enzymes (NEB). The ligation was performed with a vector: insert ratio of 1:6 using T4 ligase (NEB). Successful insertion of the HiFi‐diaCas9 fragment was verified by PCR and sequencing.

### *LHCF* gene alignments

*LHCF* gene alignments were performed in Snap Gene (GSL Biotech) using the muscle algorithm followed by manual refinement. *LHCF* mRNA sequences were downloaded from the NCBI website.

### Target genes and PAM target site selection

Six *LHCF* genes were selected for targeted gene editing using two different CRISPR/Cas9 vectors, pKS diaCas9_sgRNA (Nymark *et al.,* 2016) and HiFi‐diaCas9_sgRNA. Two different sgRNAs called LHCF multiple PAM1 and LHCF multiple PAM2 (LHCF mPAM1 and LHCF mPAM2) were designed so that both sgRNAs could potentially target the same six *LHCF* genes. Both the LHCF mPAMs had 100% match in the coding sequence of *LHCF1*, *LHCF3* and *LHCF4* and high homology to *LHCF2* (1 bp mismatch), *LHCF5* (1–2 bp mismatches), and *LHCF11* (2–3 bp mismatches). In addition, there was a 2 bp mismatch between LHCF mPAM2 and *LHCF9,* whereas LHCF mPAM1 could not target *LHCF9*. On‐ and off‐target target sites for the *LHCF* genes and other genomic loci were predicted as described previously using an in‐house PAM motif search tool (Nymark *et al.,* 2016) and verified using a web bioinformatic tool called CRISPOR (http://crispor.tefor.net/). Predicted target sites are available in Table S1. Ligation of adapters for the targets of interest was cloned into the sgRNA module of both vectors as described as in the protocol for CRISPR/Cas9 gene editing in *P. tricornutum* (Nymark *et al.,* 2017). Oligonucleotides used in this study can be found in Table S2.

### Delivery of plasmids by biolistic bombardment

Axenic *P. tricornutum* cells (CCMP2561) were co‐transformed with CRISPR/Cas9 plasmid (pKS diaCas9_sgRNA or HiFi‐diaCas9_sgRNA) in combination with pAF6 plasmid containing a selection marker conferring resistance to zeocin by biolistic bombardment as described previously (Nymark *et al.,* 2017). A total of 2.5 µg of each plasmid was used for biolistic transformation. Transformed colonies appeared on selection plates (50% Sea Water (SW), f/2 nutrients, 1% agar plates, 100 μg/mL zeocin) after 3–5 weeks of incubation. Transformed colonies were transferred to new selection plates to enable screening for colonies containing mutated cells and for maintenance of the cells. An overview of the process for mutant screening and isolation of pure secondary or tertiary colonies is shown in Figure S8.

### Mutant screening

Zeocin‐resistant colonies were screened for the presence of the Cas9 gene by PCR amplification. Colonies that were positive for a fragment of the Cas9 plasmid were additionally screened for mutations in *LHCF1* by amplifying the DNA region spanning the LHCF1 mPAM1 or mPAM2 target sites by PCR. The size and sequence of the PCR products were investigated by 1% agarose gel electrophoresis and Sanger sequencing as previously described in Nymark *et al*. (2016, 2017). Primary colonies found to contain cells with mutations in the *LHCF1* gene were selected for further investigations. Primary mutant colonies/cultures will usually contain a mixture of both WT cells and cells with a variety of indels at the target region. Diluted cell cultures (approx. 300 cells/ml) originating from primary colonies containing cells with confirmed mutations in the *LHCF1* gene were therefore spread onto selection plates to isolate single cells. Colonies originating from individual cells were screened for targeted DNA editing of *LHCF1‐5* and *LHCF11* as described above. PCR products flanking target and putative off‐target regions were amplified using primer pairs listed in Table S2. Polymorphisms present in the *LHCF* target genes were used to differentiate between the alleles, and to verify whether one or both alleles had been amplified, sequenced and edited. The *Phatr2_28684* gene (XP_002181559) was co‐amplified with the target genes as a positive control.

### Strains and growth conditions

Two lines confirmed to be quadruple (*lhcf* mPAM1 6.1.11) or quintuple (*lhcf* mPAM1 15.1) *lhc* KOs and displaying a change in coloration compared to WT were selected for phenotypic investigations. Both cell lines were created using the LHCF mPAM1 sgRNA. WT cells and *lhcf* KO mutants were grown as described previously (Nymark *et al.,* 2009, 2016). In short, cells were maintained in f/2 liquid medium (Guillard, 1975) at 22°C under white fluorescent light (60 µmol photons/m^2^/s), with shaking (150 rpm) without aeration at a photoperiod of 16 h light:8 h dark. Before performing LL (˜50 μmol photons/m^2^/s) and HL (˜280 μmol photons/m^2^/s) experiments, the WT and *lhcf* mutants were acclimated to the respective light conditions for two weeks. The growth media were refreshed every second day before experiments were conducted to keep the cells in the exponential phase. Three biological replicates were used for each cell line and all experiments.

### Growth rates

Growth rates were calculated for the different cell lines in LL and HL by daily cell counting using a NovoCyte flow cytometer (ACEA Biosciences). The flow cytometer’s 488 nm laser was used for excitation of the samples, and the autofluorescence from chlorophyll was collected on a detector with a 675/30 nm bandpass filter.

### Measurements of maximum quantum yield of PSII photochemistry

The maximum quantum yield of PSII photochemistry (Φ_PSIImax_) was calculated from *F_v_*/*F_m_* as described previously (Nymark *et al.,* 2009) using an AquaPen‐C (Photon System Instruments). All samples were incubated 3 min in darkness prior to performing measurements.

### Pigment analyses

Pigment content per cell in the WT and *lhc* KO lines in LL and HL were analysed by HPLC using a Hewlett‐Packard HPLC 1100 Series system equipped with a diode array detector (DAD) and a Waters Symmetry C8 column. HPLC analyses were performed as described in Rodriguez et al. (2006). Pigments were detected by absorbance at 440 nm and identified by the DAD in the wavelength range of 350–750 nm (1.2 nm spectral resolution). The specific extinction coefficient (L/g/cm), provided by Humphrey and Jeffrey (1997), was used together with the extraction volume, the filtration volume and the sample injection volume to calculate the pigment concentrations. The pigment concentrations were presented as mol of Fuco per mol of Chl *a* (Jeffrey *et al.,* 1997).

### Protein isolation and Western blot analysis

WT and the two multiple *lhc* KO mutant cultures acclimated to LL and HL were harvested by filtration (Durapore Membrane Filters, pore size 0.6 μm; Merck Millipore). Filters with harvested cells were placed in 2 mL tubes (Sarstedt), and cells were re‐suspended in 1 mL f/2 medium by vortexing for 10 s. The filters were removed from the tubes before centrifugation at 16 000 ***g*** for 1 min at room temperature. The supernatants were discarded, and the resulting pellets were flash frozen in liquid nitrogen and stored at −80°C. A pre‐cooled 5 mm steel bead (QIAGEN) was added to each tube containing frozen cell pellets, and the cell pellets were mechanically broken in two steps using the TissueLyser II system (QIAGEN). In the first step, tubes containing cell pellets were placed in a precooled (−80°C) adapter set, and the cell material was disrupted for 2 min at 25 Hz. In the second step, the tubes were transferred to a room temperature adapter set after adding 700 μl lysis buffer (50 mM Tris, pH 6.8, 2% (w/v) SDS) and disrupted for 8 min at 25 Hz (Juhas et al., 2014). Lysates were centrifuged (30 min, 100***g***, 4°C) to remove insoluble material. The supernatants were transferred to new tubes, and the total protein concentrations were determined using the DC Protein Assay kit (BioRad) following the manufacturer’s instructions.

Western blot analyses were performed on total protein extract for the detection of LHCF proteins. A total of 10 μg of the total protein extracts were loaded onto the gel (15% sodium dodecyl sulphate (SDS)–polyacrylamide separation gel). Proteins were detected with the following antibodies: anti‐D1 (AS05 084 Agrisera; 1:20 000) and anti‐LHCF1‐11 (gift from Prof. C. Büchel, University of Frankfurt, Germany (Juhas et al., 2014); 1:1000). Primary antibody incubation was performed overnight at 4°C for both antibodies. Polyclonal Goat Anti‐Rabbit Immunoglobulins/Biotinylated (Dako) was used as a secondary antibody with an incubation time of 2 h at room temperature (RT), followed by incubation with Horseradish Peroxidase Streptavidin (Vector Laboratories) for 1 h at RT. Protein‐antibody cross‐reactions were visualized with SuperSignal West Pico PLUS Chemiluminescent Substrate (Thermo Scientific) and documented with a G:BOX ChemiXRQ gel doc system (Syngene).

### Quantitative real‐time PCR (qRT‐PCR) for determination of gene expression level

Around 5 × 10^6^ cells were collected by filtration (DVPP, 0.65 µm, Merck Millipore) from 40 ml of culture. Cells from filter papers were re‐suspended in 1 ml of f/2 media and centrifuged for 1 min at 16 000 ***g***. Total RNA was extracted from WT, *lhcf* mPAM1 15.1 and *lhcf* mPAM1 6.1.11 lines using Spectrum Plant Total RNA Kit (Sigma‐Aldrich) according to the manufacturer’s instructions. RNA quantification was done by using a NanoDrop Spectrophotometer (ND 1000, NanoDrop technologies). Complementary DNA (cDNA) was synthesized from 1000 ng of total RNA using QuantiTect Reverse Transcription Kit (Qiagen) following the manufacturer’s instructions.

Quantitative RT‐PCR was performed using LightCycler 480 SYBR green I Master kit (Roche) and a LightCycler 96 instrument (Roche). PCR efficiencies were calculated for each sample using LinReg PCR 11.1 software (Ramakers *et al.,* 2003). *Phatr2_28684* and *Phatr2_2418635* genes were used as internal reference genes for normalization of the relative expression of genes (Alipanah *et al.,* 2015). Calculations of the relative gene expression were performed using the qRT‐PCR analysis software qbase+ version 3.2 (Biogazelle, www.qbaseplus.com). The mean of the individual biological replicates (*n* = 3) was calculated and used to determine the ratio of gene expression. Relative expression of genes was normalized to WT. Primers used for the qRT‐PCR can be found in Table S3.

## Conflict of interest

The authors declare no conflict of interests.

## Author contributions

A.S., M.N., A.M.B. and P.W. conceived and planned the experiments. A.S. and P.W. designed and produced the HiFi Cas9 vector. P.W. and A.M.B. supervised and provided resources. A.S., S.F., T.S. and M.N. generated and analysed the LHCF mutant lines, including growth, pigment and Western blot analyses. A.S. and M.N. drafted the manuscript. P.W. and A.M.B. revised the manuscript. All authors have reviewed and approved the final manuscript.

## Supporting information

**Figure S1** DNA sequence alignment of LHCF1‐10 from *Phaeodactylum tricornutum* and LHCF_multi PAM target sequences.
**Figure S2** Comparison of Cas9‐editing efficiency in cells transformed with vectors expressing diaCas9 or HiFi‐diaCas9.
**Figure S3** PCR amplification of target (LHCF1, LHCF3, LHCF4) and off‐target (LHCF2, LHCF5, LHCF11) regions, genomic sequence and LHCF3‐4 fusion protein.
**Figure S4** Relative mRNA expression of WT, *lhcf* mPAM1 15.1 and *lhcf* mPAM1 6.1.11 mutants.
**Figure S5** Genotype and phenotype of *lhcf* mPAM1 1.10.
**Figure S6** Cell growth and maximum quantum yield of photosystem II in WT and *lhcf* mPAM1 15.1 and 6.1.11 KO lines.
**Figure S7** Comparison of on‐target and off‐target editing between standard diaCas9 (black) and HiFi‐diaCas9 (light grey).
**Figure S8** An overview of the process for mutant screening and isolation of pure secondary and tertiary clones.
**Table S1** Overview of DNA sequences targeted by LHCF mPAM1 and LHCF mPAM2 sgRNAs.
**Table S2** Oligos and primers used in the experiment.
**Table S3** Primers used to analyse gene expression in the experiment.
