# Supplementary material for: Simultaneous knockout of multiple LHCF genes using single sgRNAs and engineering of a high‐fidelity Cas9 for precise genome editing in marine algae
Source: Plant Biotechnol J. 2021 Apr 9;19(8):1658–69. doi: 10.1111/pbi.13582 (PMC8384595; doi:10.1111/pbi.13582)
Supplement: Supplementary file 1 — Figure S1 DNA sequence alignment of LHCF1‐10 from Phaeodactylum tricornutum and LHCF_multi PAM target sequences. Figure S2 Comparison of Cas9‐editing efficiency in cells transformed with vectors expressing diaCas9 or HiFi‐diaCas9. Figure S3 PCR amplification of target (LHCF1, LHCF3, LHCF4) and off‐target (LHCF2, LHCF5, LHCF11) regions, genomic sequence and LHCF3‐4 fusion protein. Figure S4 Relative mRNA expression of WT, lhcf mPAM1 15.1 and lhcf mPAM1 6.1.11 mutants. Figure S5 Genotype and phenotype of lhcf mPAM1 1.10. Figure S6 Cell growth and maximum quantum yield of photosystem II in WT and lhcf mPAM1 15.1 and 6.1.11 KO lines. Figure S7 Comparison of on‐target and off‐target editing between standard diaCas9 (black) and HiFi‐diaCas9 (light grey). Figure S8 An overview of the process for mutant screening and isolation of pure secondary and tertiary clones. Table S1 Overview of DNA sequences targeted by LHCF mPAM1 and LHCF mPAM2 sgRNAs. Table S2 Oligos and primers used in the experiment. Table S3 Primers used to analyse gene expression in the experiment. [file PBI-19-1658-s001.pdf]

# Simultaneous knock-out of multiple *LHCF* genes using single sgRNAs and engineering of a high fidelity Cas9 for precise genome editing in marine algae.

Sharma AK[1], Nymark M[1], Flo S[1,2], Sparstad T[1], Bones AM[1], Winge P[1].

1. Cell, Molecular biology and Genomics Group, Department of Biology, Norwegian University of Science and Technology, N-7491 Trondheim, Norway.
2. Present address : The University Centre in Svalbard, UNIS, N-9171 Longyearbyen, Norway

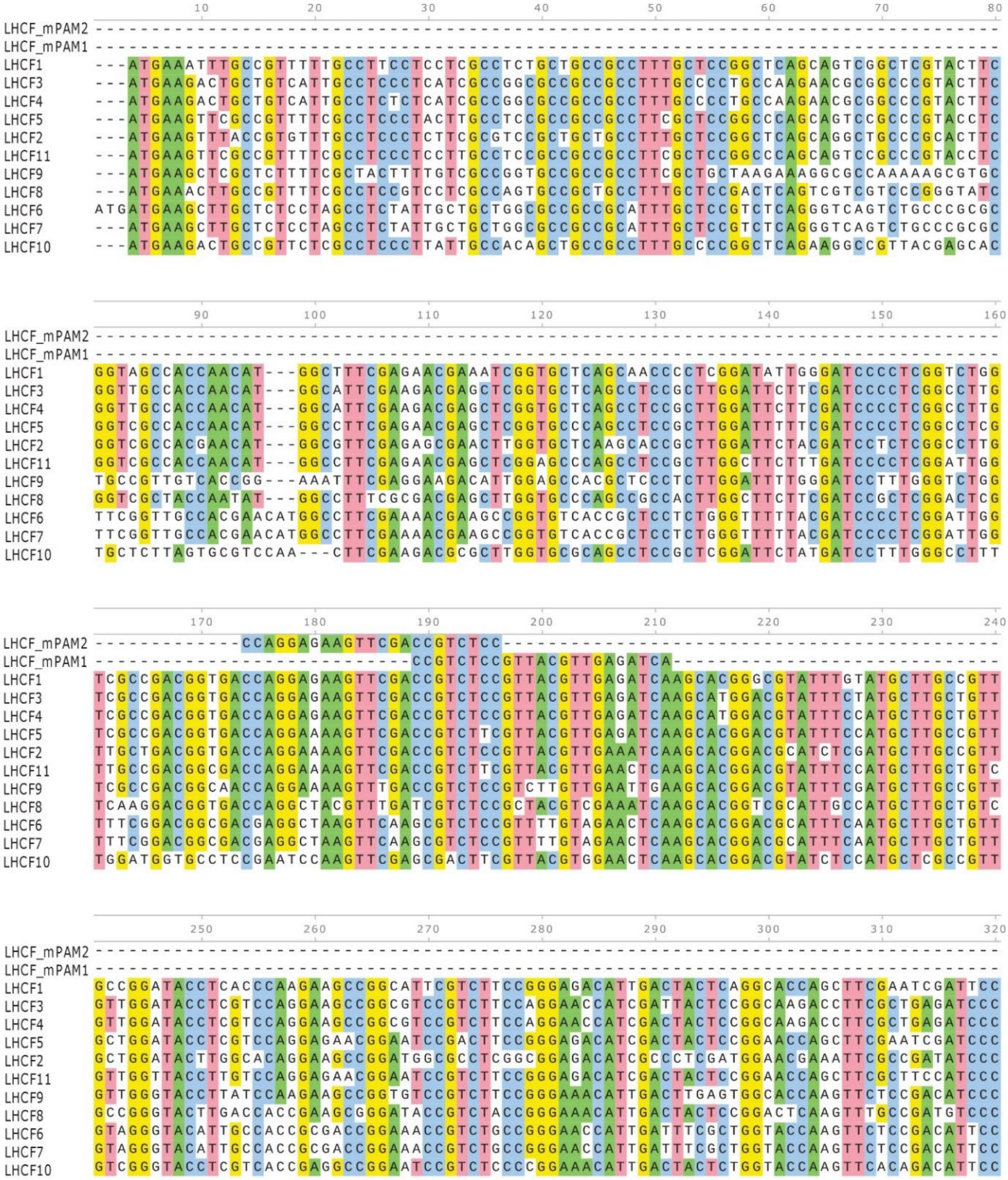
10
20
30
40
50
60
70
80
LHCF_mPAM2
LHCF_mPAM1
LHCF1
LHCF3
LHCF4
LHCF5
LHCF2
LHCF11
LHCF9
LHCF8
LHCF6
LHCF7
LHCF10
90
100
110
120
130
140
150
160
170
180
190
200
210
220
230
240
250
260
270
280
290
300
310
320

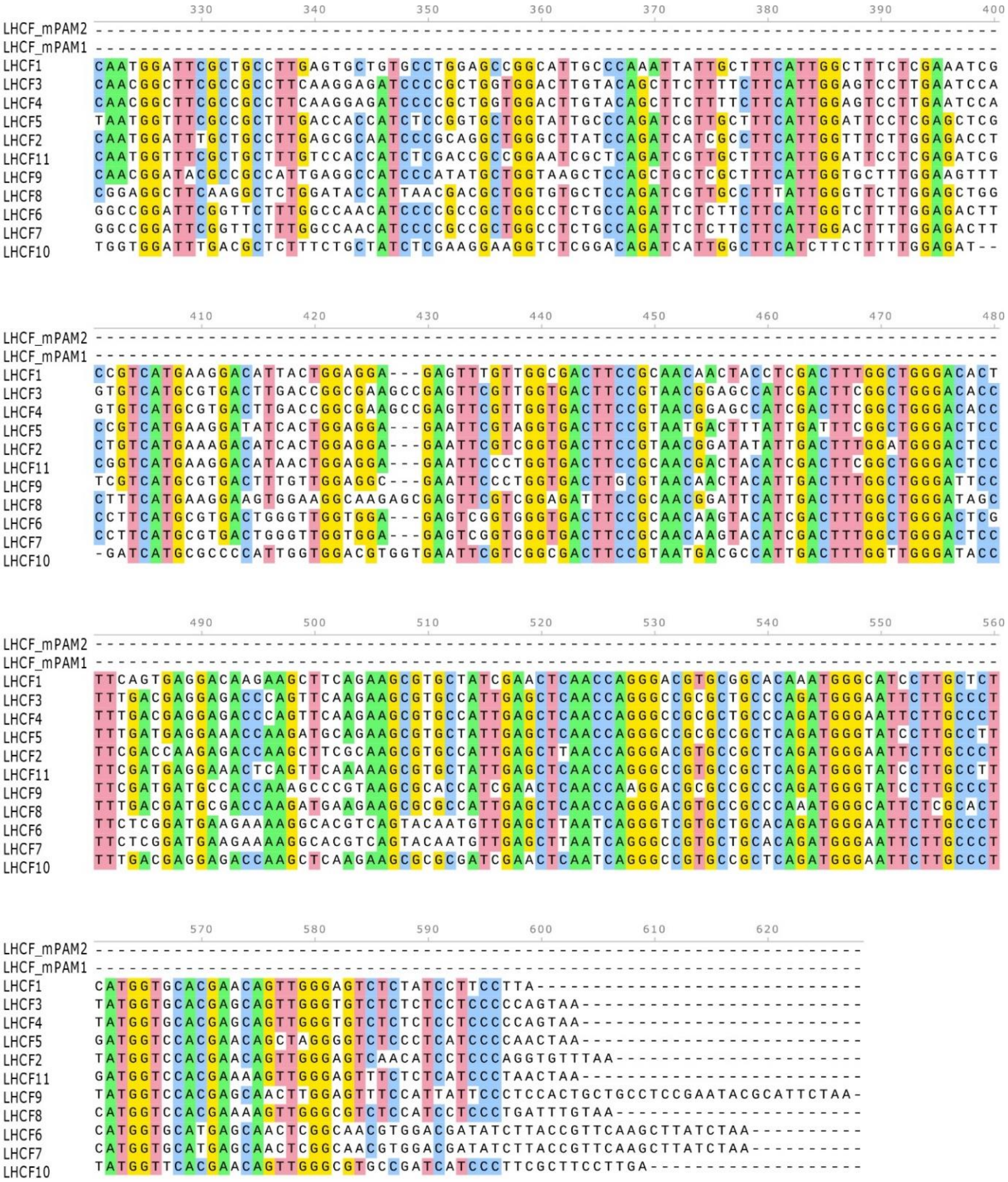


**Figure S1: DNA sequence alignment of *LHCF1-10* from *Phaeodactylum tricornutum* and LHCF_multi PAM target sequences**. Alignments were made in Snap Gene (GSL Biotech) using the Muscle algorithm.

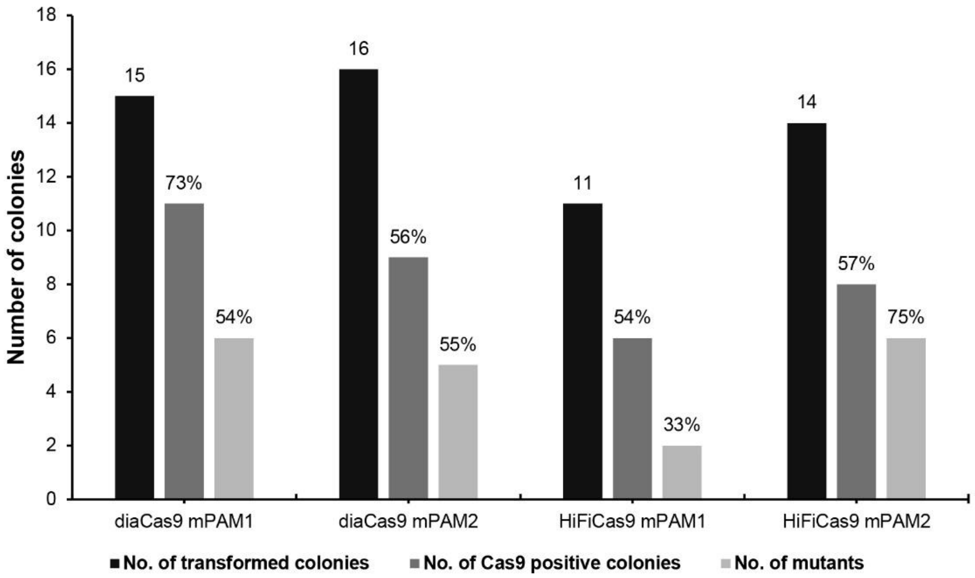


**Figure S2: Comparison of Cas9-editing efficiency in cells transformed with vectors expressing diaCas9 or HiFi-diaCas9.** Total number of transformed colonies, primary colonies confirmed to contain a Cas9 fragment and number of Cas9-positive colonies edited at the *LHCF1* target sites are presented. The fraction of Cas9 positive colonies relative to the total number of transformants, and the fraction of colonies containing cells with mutations in *LHCF1* relative to the number of Cas9-positive colonies are indicated above the bars.

A.

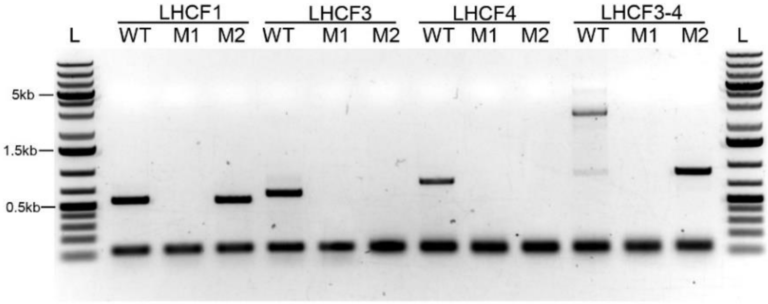


B.

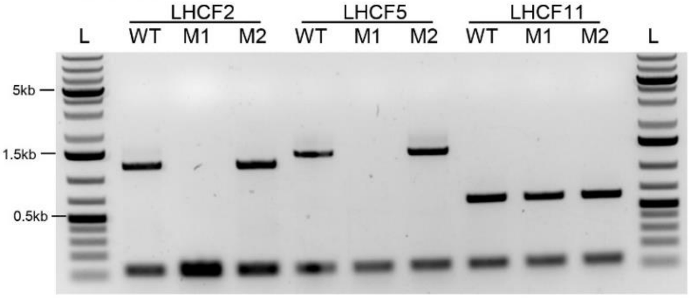


C. > LHCF3-4 C6.1.11

GCTTGAACACGATCCTTGTGAAAGGCTGACGTTGCGCGAAAAAACAGCCCGCCGATTTCTTTCCTTCTTT
CTCGTCGTAAACTATATACTTTCATAATCTCTGTTTGAGTTTACCAACAACACATATATACATTTCGACA
AAATGAAGACTGCTGTCATTGCCTCCCTCATCGCCGGCGCCGCCGCCTTTGCCCCTGCCAAGAACGCGGC
CCGTACTTCGGTTGCCACCAACATGGCATTCGAAGACGAGCTCGGTGCTCAGCCTCCGCTTGGATTCTTC
GATCCCCTCGGCCTTGTCGCCGACGGTGACCAGGAGAAGTTCGA**CCG**TCTCCGTTACGTT**GAAATCAAGC**
**ACGGACGCATCTCG**ATGCTTGCTGTTGTTGGATACCTCGTCCAGGAAGCCGGCGTCCGTCTTCCAGGAAC
CATCGACTACTCCGGCAAGACCTTCGCTGAGATCCCCAACGGCTTTGCCGCCTTCAAGGAGATCCCCGCT
GGTGGACTTGTACAGCTTCTTTTCTTCATTGGAGTCCTTGAATCCAGTGTCATGCGTGATTTGACCGGCG
AGGCCGAGTTCGTTGGTGACTTCCGTAACGGAGCCATCGACTTCGGCTGGGACACCTTTGACGAGGAGAC
CCAGTTCAAGAAGCGTGCCATTGAGCTCAACCAGGGCCGCGCTGCCCAGATGGGAATTCTTGCCCTTATG
GTGCACGAGCAGTTGGGTGTCTCCCTCCTCCCCCAGTAATTTTGTTACATTTACTGACTTCAAGGAGTCG
AGGAATCGATACTGCCGTCGTTTCCAGGATCCGAGGTTTCATAAACTCTGTTAACGTTATAGAAACAGAC
TTACCTCTCCTACGCCATTCACGTAATATTCGC

D. >LHCF3-4 fusion protein

MKTAVIASLIAGAAAFAPAKNAARTSVATNMAFEDELGAQPPLGFFDPLGLVADGDQEKFDRLRYVEIKH
GRISMLAVVGYLVQEAGVRLPGTIDYSGKTFAEIPNGFAAFKEIPAGGLVQLLFFIGVLESSVMRDLTGE
AEFVGDFRNGAIDFGWDTFDEETQFKKRAIELNQGRAAQMGILALMVHEQLGVSLLPQ

**Figure S3: PCR amplification of target (LHCF1, LHCF3, LHCF4) and off-target (LHCF2, LHCF5, LHCF11) regions, genomic sequence and LHCF3-4 fusion protein. A-B)** Genomic regions predicted to be targeted for Cas9-induced editing in *P. tricornutum* wild type (WT), *lhcf* mPAM1 15.1 (M1) and *lhcf* mPAM1 6.1.11 (M2) lines were PCR amplified using primer pairs listed in supplementary Table S2. LHCF3-4 refers to PCR product amplified using LHCF3 forward and LHCF4 reverse primer pair. Phatr2_28684 gene (lower band in gel) was co-amplified as a positive control. 1kb plus DNA ladder (L; Thermo Fisher) was used for relative size comparison. **C)** Genomic sequence of *lhcf* mPAM1 6.1.11, gene fusion of LHCF3 and LHCF4, (see agarose gel separation of PCR fragment in S3A). Red letters LHCF3 5’ region and LHCF4 3’ region. Start codon and stop codon shown in underlined letters. The 24 bp LHCF2 patch insertion is shown in blue. PAM1 site shown by underlined letters. **D)** The LHCF3-4 fusion protein is identical to LHCF3 / LHCF4. LHCF3 and LHCF4 are identical at amino acid sequence level.

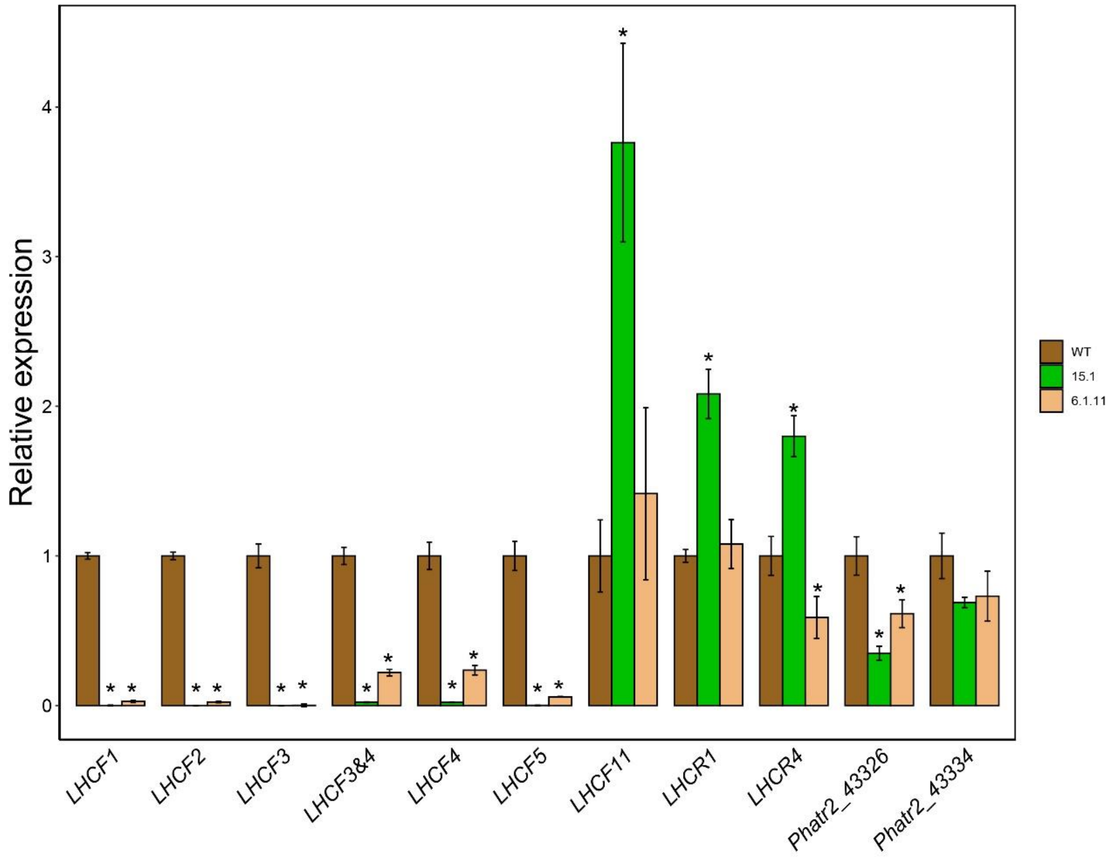


**Figure S4: Relative mRNA expression of WT, *lhcf* mPAM1 15.1 and *lhcf* mPAM1 6.1.11 mutants.** The histograms show relative gene expression compared with WT cells. Each data point represents the mean value (n = 3) and error bars represent standard deviations (SD). On-target genes are *LHCF1*, *LHCF3* and *LHCF4*. Off-target genes are *LHCF2*, *LHCF5* and *LHCF11*. Genes upstream of *LHCF1* is *Phatr2_43334* and downstream of *LHCF4* is *Phatr2_43326*. *LHCR1* and *LHCR4* encode red algal-like LHC proteins associated mainly with PSI. Gene expression of *LHCF3* and *LHCF4* were analysed using C-terminal gene specific primers LHCF3 Rw and LHCF4 Rw, respectively. LHCF3 & 4 were analysed using primers pairs that could recognize both LHCF3 and LHCF4. For primer details, see Table S3. Asterisks indicate significant difference (*, $P < 0.05$) compared with WT. Data are expressed as means ± SD (n = 3).

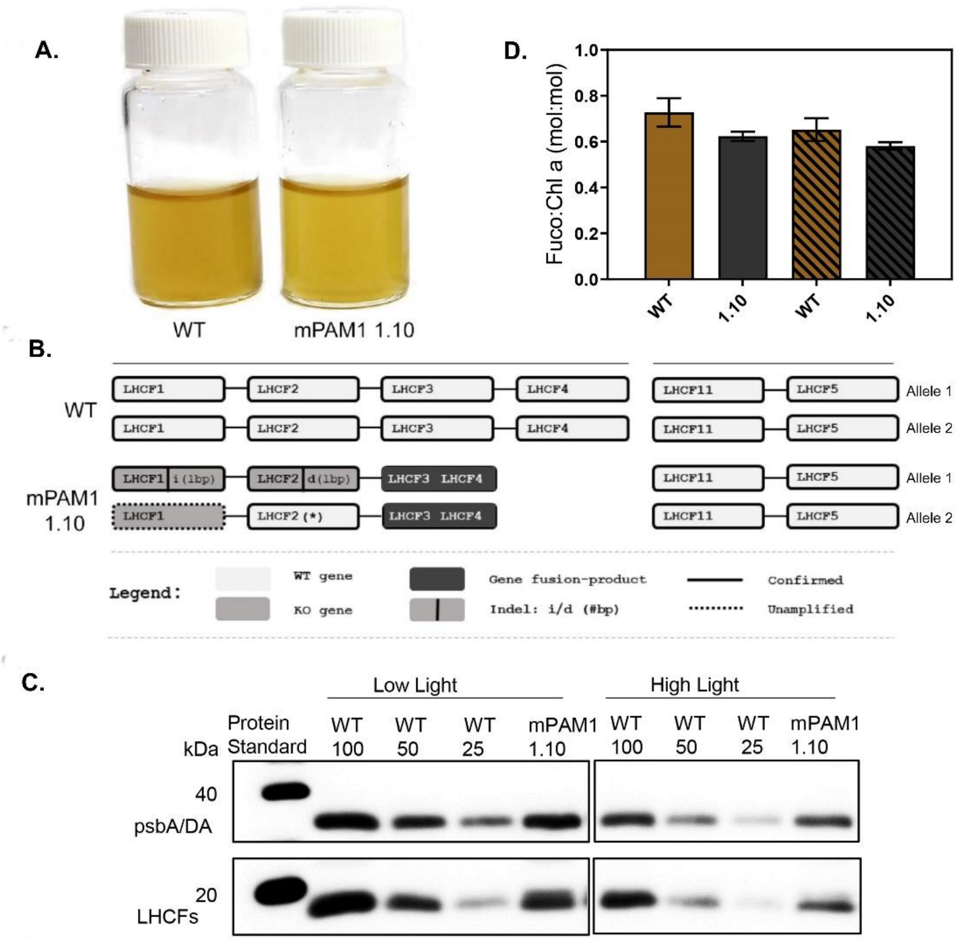


**Figure S5: Genotype and phenotype of *lhcf* mPAM1 1.10. A)** Phenotypes of high light (HL, 280 µmol photons $m^{-2}s^{-1}$) and low light (LL, 50 µmol photons $m^{-2}s^{-1}$) acclimated *P. tricornutum* WT and mPAM1 1.10 cultures. Both cell cultures were adjusted to equal cell density (10 million $mL^{-1}$) and imaged by iPhone 6s. **B)** Schematic overview of mutated *LHCF* genes in mPAM1 1.10. and WT genes are white. Knockout (KO) genes are light-grey and annotated by indel (i/d) and the base length of the indel (#bp) if known. In-frame gene fusion-products are labelled dark grey. Confirmed DNA sequences are labelled with a solid border. Target region not possible to amplify by PCR have dashed borders. (*) denotes a re-edited copy of *LHCF2* in cell line mPAM1 1.10, in which a1 bp deletion was repaired to a perfect WT sequence. **C)** Western blot analysis of LHCFs proteins from WT and mPAM1 1.10 mutant acclimated to LL and HL conditions. *P. tricornutum* WT and mPAM1 mutant 1.10 were grown for two weeks in HL and LL before harvesting and isolation of total protein. Proteins separated on an SDS-PAGE gel were blotted on a nitrocellulose membrane, and polyclonal rabbit antibodies were used to target LHCFs, and photosystem II D1-protein (psbA/D1). MagicMarkTM XP Western Protein standards report the protein mass (in kDa). D) Pigment level in WT and mPAM1 1.10 lines. WT and mPAM1 1.10 lines acclimated to HL (280 µmol photons $m^{-2}s^{-1}$) and LL (50 µmol photons m-2s-1) intensities. Fuco per Chl *a* (Fuco:Chl *a*) ratios are presented as the mean of n replicates of the relative unit mol:mol, with the standard error of the mean (±SEM, nWT=9, n1.10=4).

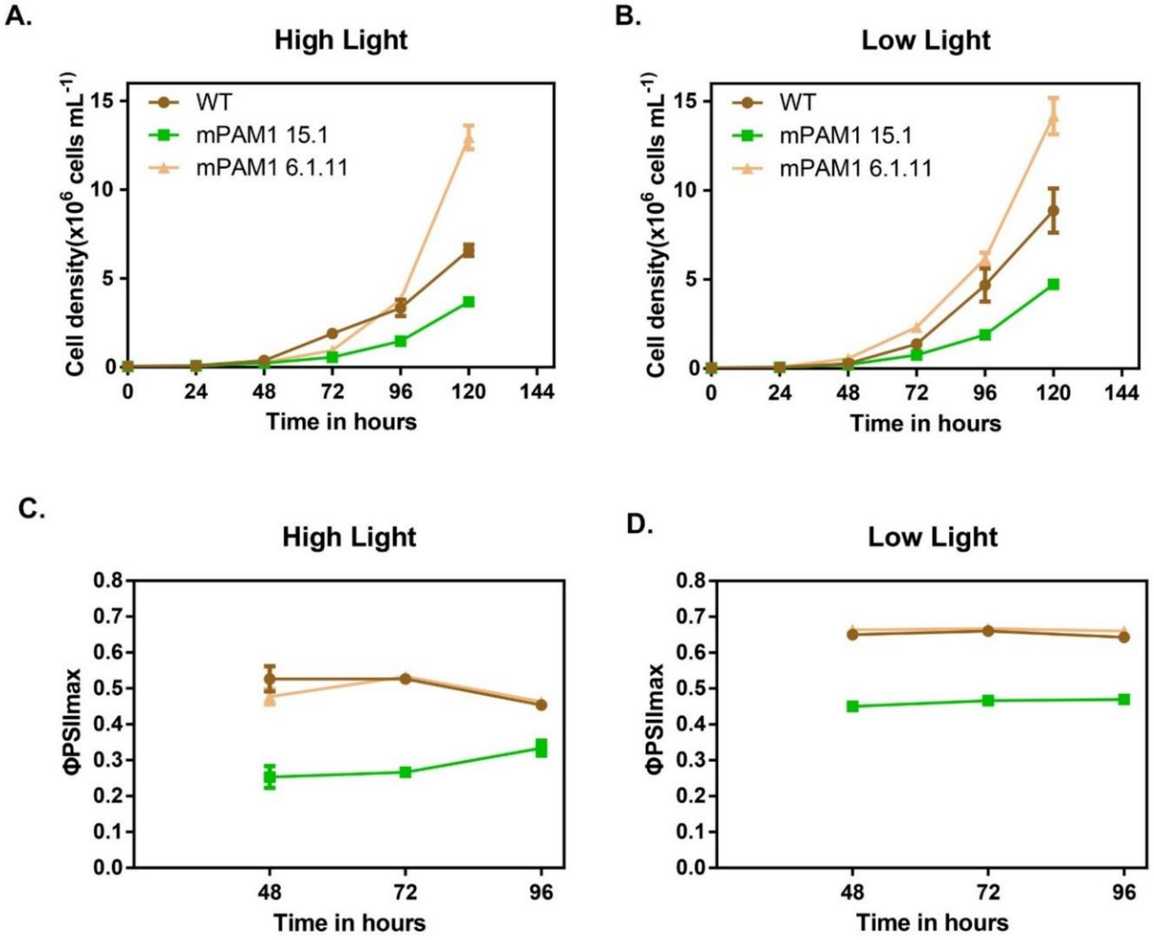


**Figure S6: Cell growth and maximum quantum yield of photosystem II in WT and *lhcf* mPAM1 15.1 and 6.1.11 KO lines.** Growth curves for WT and multiple *lhcf* KO mutants acclimated to A) high light (HL; 280 µmol photons $m^{-2}s^{-1}$) and B) low light (LL; 50 µmol photons $m^{-2}s^{-1}$) intensities. Maximum quantum yield of photosystem II at C) HL and D) LL. Results are presented as a mean of three biological replicates with ±SD.

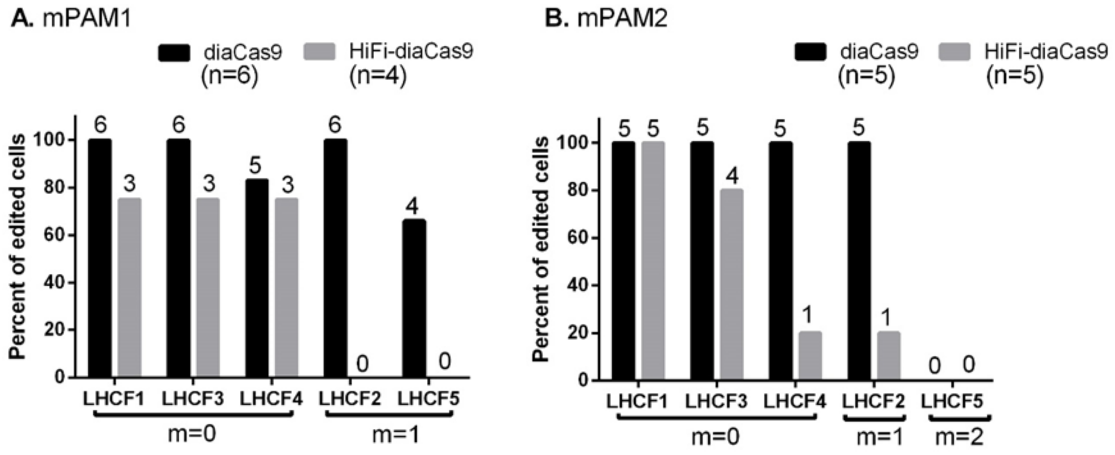


**Figure S7: Comparison of on-target and off-target editing between standard diaCas9 (black) and HiFi-diaCas9 (light grey).** The percentage of screened mutants in which editing events occurred in LHCF1-5 using A) LHCF mPAM1 sgRNA and B) LHCF mPAM2 sgRNA. Both detected indels and unamplified target regions are considered editing events. Editing does not distinguish between mono- or bi- allelic mutation. The number of base-pairing mismatches (m) between sgRNA and target sites are indicated below the gene names. Number above the bars represent number of colonies edited. Total number (n) of mutants screened is indicated below each of the Cas9s.

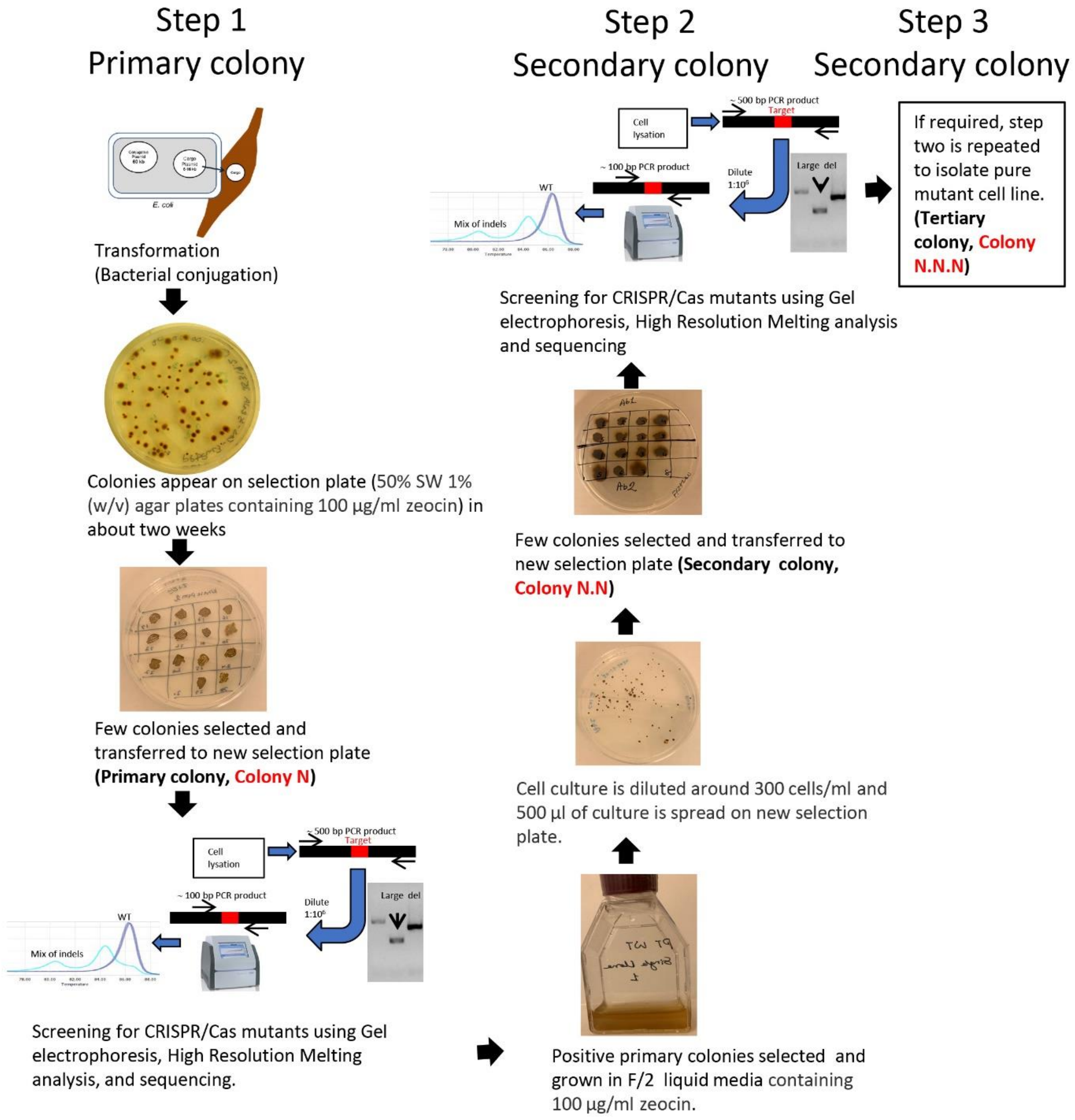


**Figure S8: An overview of the process for mutant screening and isolation of pure secondary and tertiary clones.**

**Table S1. Overview of DNA sequences targeted by LHCF mPAM1 and LHCF mPAM2 sgRNAs.** Mismatched nucleotides are labelled red. PAM-sequences (NGG) are underlined. Target sequences are described with their number of mismatch count against the sgRNAs, DNA positive (+) or negative (-) strands, chromosomal location (Chr.), locus description and accession IDs corresponding to *P. tricornutum* annotation Phatr3, available at Ensembl.

| **LHCF mPAM1 targets (5'-3')** | **Mismatch count** | **Strand** | **Chr.** | **Locus description** | **Accession ID (Phatr3)** |
|---|---|---|---|---|---|
| TGATCTCAACGTAACGGAGACGG | 0 | + | 2 | Exon: *LHCF1* | *Phatr3_J18049* |
| TGATCTCAACGTAACGGAGACGG | 0 | + | 2 | Exon: *LHCF3* | *Phatr3_J50705* |
| TGATCTCAACGTAACGGAGACGG | 0 | + | 2 | Exon: *LHCF4* | *Phatr3_J50705* |
| TGATTTCAACGTAACGGAGACGG | 1 | + | 2 | Exon: *LHCF2* | *Phatr3_J25172* |
| TGATCTCAACGTAACGAAGACGG | 1 | + | 24 | Exon: *LHCF5* | *Phatr3_J30648* |
| TGAGTTCAACGTAACGAAGACGG | 3 | + | 24 | Exon: *LHCF11* | *Phatr3_J51230* |
| CCACCTCAACGTAACCGAGACGG | 4 | - | 4 | Exon: *CDKC1* | *Phatr3_EG02630* |
| TGAGTACAACGTCACGGAGAGGG | 4 | + | 5 | Pred. Protein | *Phatr3_J44831* |
| TTCTCGCAACGTAACGGAAAAGG | 4 | - | 9 | Pred. Protein | *Phatr3_J12752* |
| | | | | | |
| **LHCF mPAM2 targets (5'-3')** | | | | | |
| GGAGACGGTCGAACTTCTCCTGG | 0 | + | 2 | Exon: *LHCF1* | *Phatr3_J18049* |
| GGAGACGGTCGAACTTCTCCTGG | 0 | + | 2 | Exon: *LHCF3* | *Phatr3_J50705* |
| GGAGACGGTCGAACTTCTCCTGG | 0 | + | 2 | Exon: *LHCF4* | *Phatr3_J50705* |
| GGAGACGGTCGAACTTTTCCTGG | 1 | + | 2 | Exon: *LHCF2* | *Phatr3_J25172* |
| GAAGACGGTCGAACTTTTCCTGG | 2 | + | 24 | Exon: *LHCF5* | *Phatr3_J30648* |
| GAAGACGGTCGAACTTTTCCTGG | 2 | + | 24 | Exon: *LHCF11* | *Phatr3_J51230* |
| GGAGACGGTCAAACTTTTCCTGG | 2 | + | 20 | exon: *LHCF9* | *Phatr3_EG00427* |
| TGTGACGGCCGAACATCTCCTGG | 4 | + | 18 | Pred. Protein | *Phatr3_J39147* |
| | | | | | |

**Table S2. Oligos and primers used in the experiment.**

| Name of primer | Sequence of primer (5'-3') | Amplicon size (bp) | Comment |
|---|---|---|---|
| LHCF mPAM1 F | TCGATGATCTCAACGTAACGGAGA | | LHCF mPAM1 forward oligo |
| LHCF mPAM1R | AAACTCTCCGTTACGTTGAGATCA | | LHCF mPAM1 reverse oligo |
| LHCF mPAM2F | TCGAGGAGACGGTCGAACTTCTCC | | LHCF mPAM2 forward oligo |
| LHCF mPAM2R | AAACGGAGAAGTTCGACCGTCTCC | | LHCF mPAM2 reverse oligo |
| LHCF mPAM1 F | TCGATGATCTCAACGTAACGGAGA | 544 | Verifying LHCF mPAM1 insert into plasmid |
| M13 Rev | CAGGAAACAGCTATGAC | | |
| LHCF mPAM2F | TCGAGGAGACGGTCGAACTTCTCC | 544 | Verifying LHCF mPAM2 insert into plasmid |
| M13 Rev | CAGGAAACAGCTATGAC | | |
| LHCF1F | TGTTTTGACTGCAAGATCAGC | 555 | PCR amplification or sequencing of LHCF1 |
| LHCF1R | CGGAAGTCGCCAACAAACTCT | | |
| LHCF2F | CAAATCCTGGTTGAACACGTA | 1223 | PCR amplification or sequencing of LHCF2 |
| LHCF2R2 | AGTTAGTCCCTTCACCTTTGGC | | |
| LHCF3F | GCTTGAACACGATCCTTGTGA | 638 | PCR amplification or sequencing of LHCF3 |
| LHCF3R | TGAACTGGGTCTCCTCGTCA | | |
| LHCF4F | GCCCTCATAATCTCTGTTAGA | 786 | PCR amplification or sequencing of LHCF4 |
| LHCF4R2 | GCGAATATTACGTGAATGGCG | | |
| LHCF5F | AGTGCTATGCCTGAAATGTGC | 688 | PCR amplification or sequencing of LHCF5 |
| LHCF5R | TCTAACCGGAGGAACATCAAC | | |
| LHCF11F | ACACCACCTGGATCTTCACAT | 618 | PCR amplification or sequencing of LHCF11 |
| LHCF11R | CAACTTTTCGTGGACCATCAA | | |
| LHCF9F | CCGATCTTTCCGCAACCTTTATC | 689 | PCR amplification or sequencing of LHCF11 |
| LHCF9R | CGTCTAAACATCGTTAGGTATTG | | |
| LHCF3F | GCTTGAACACGATCCTTGTGA | 2605 | PCR amplification or sequencing of LHCF3-4 |
| LHCF4R2 | GCGAATATTACGTGAATGGCG | | |
| dCas9 F1 | GAAGTACTCGATTGGATTGGAC | 817 | Verification of Cas9 fragments |
| dCas9R1 | GAGGTCGTCGTCATATGTGTCT | | |
| qPCRphat_28684F | TGCGCTCGACACCATTTCAA | 137 | Co-amplified as Control during PCR |
| qPCRphat_28684R | GCTCCTTTGAAGATTGGACG | | |
| | | | |

**Table S3. Primers used to analyse gene expression in the experiment.**

| Name of primer | Sequence of primer (5’-3’) | Amplicon size (bp) | Comment |
|---|---|---|---|
| q43334 Fw | GTCATCTTGAGGATCCGGTA | 221 | To check expression of *LHCF1* upstream gene*, Phatr2_43334* |
| q43334 Rw | ACGCTGTATTGGATGGCTAC | | |
| qLHCF1 Fw | CGGATATTGGGATCCCCTCGGT | 124 | To check expression of *LHCF1 gene* |
| qLHCF1 Rw | TTGGGTGAGGTATCCGGCAACG | | |
| qLHCF3 Fw | CAAGGAGTCGAGGAATCGAT | 105 | To check expression of *LHCF3 gene* |
| qLHCF3 Rw | GCAGGAGAGGTATGTCTGAT | | |
| qLHCF3 Fw | CAAGGAGTCGAGGAATCGAT | 60 | To check expression of *LHCF4 gene* |
| qLHCF4 Rw | CAGAGTTTATGAAACCTCGG | | |
| q43326 Fw | CAGAATCGGAAGATGAGTGG | 131 | To check expression of *LHCF4 down*stream gene*, Phatr2_43326* |
| q43326 Rw | TCCTAGACACATCTGTTGGA | | |
| qLHCF11 Fw | CCGCAACGACTACATCGACT | | To check expression of *LHCF11 gene* |
| qLHCF11Rw | CAACTTTTCGTGGACCATCAA | 131 | |
| qLHCF2 Fw | CCACGAACAGTTGGGAGTCAA | 109 | To check expression of *LHCF2 gene* |
| qLHCF2 Rw | CAACGCAATGCTTCGAACCAG | | |
| qLHCF5 Fw | CATCTCCGGTGCTGGTATTG | | To check expression of *LHCF5 gene* |
| qLHCF5 Rw | CATCAAAGGAGTCCCAGCCG | 140 | |
| qLHCR1 Fw | ACCTGGTAATCTTGGATTCGAC | 84 | To check expression of *LHCR1 gene* |
| qLHCR1 Rw | GATTTCCTTGAGTTGCATGGCT | | |
| qLHCR4 Fw | AAGCCAAGTATGTCGCCAGTGA | 70 | To check expression of *LHCR4 gene* |
| qLHCR4 Rw | ACCGACGTATCCTTTGAGGTTG | | |
| Phatr2_24186R | CAGCCGATCCGTCATAACTGC | 137 | Gene used as reference, Ref1(XPO1) |
| Phatr2_24186F | TCTCCGATACGGGCTTGGA | | |
| Phatr2_28684F | TGCGCTCGACACCATTTCAA | 160 | Gene used as reference, Ref 2( Exportin 1) |
| Phatr2_28684R | GCTCCTTTGAAGATTGGACG | | |
